# Epidemiological and Clinical Characteristics of Five Rare Pathological Subtypes of Hepatocellular Carcinoma

**DOI:** 10.3389/fonc.2022.864106

**Published:** 2022-04-08

**Authors:** Xiaoyuan Chen, Yiwei Lu, Xiaoli Shi, Guoyong Han, Long Zhang, Chuangye Ni, Jie Zhao, Yun Gao, Xuehao Wang

**Affiliations:** ^1^ Hepatobiliary Center, The First Affiliated Hospital of Nanjing Medical University, Nanjing, China; ^2^ Key Laboratory of Liver Transplantation, Chinese Academy of Medical Sciences, Nanjing, China; ^3^ NHC Key Laboratory of Living Donor Liver Transplantation, Nanjing Medical University, Nanjing, China; ^4^ School of Medicine, Southeast University, Nanjing, China; ^5^ Department of General Surgery, The Affiliated Jiangning Hospital of Nanjing Medical University, Nanjing, China

**Keywords:** hepatocellular carcinoma, pathological subtype, fibrolamellar carcinoma, scirrhous carcinoma, clear cell carcinoma, spindle cell carcinoma, pleomorphic carcinoma, The SEER Program

## Abstract

**Background:**

Hepatocellular carcinoma (HCC) is a highly heterogeneous tumor with several rare pathological subtypes and which is still poorly understood. This study aimed to describe the epidemiological and clinical spectrum of five rare HCC subtypes and develop a competing risk nomogram for cancer-specific survival prediction.

**Methods:**

The study cohort was recruited from the Surveillance, Epidemiology, and End Results database. The clinicopathological data of 50,218 patients histologically diagnosed with classic HCC and five rare subtypes (ICD-O-3 Histology Code = 8170/3-8175/3) between 2004 and 2018 were reviewed. The annual percent change (APC) was calculated utilizing Joinpoint regression. The nomogram was developed based on multivariable competing risk survival analyses. Akaike information criterion, Bayesian information criterion, C-index, calibration curve, and area under the receiver operating characteristic curve were obtained to evaluate the prognostic performance. A decision curve analysis was introduced to examine the clinical value of the models.

**Results:**

Despite scirrhous carcinoma, which showed a decreasing trend (APC = -6.8%, *P* = 0.025), the morbidity of other rare subtypes remained stable from 2004 to 2018. The incidence-based mortality was plateau in all subtypes during the period. Clear cell carcinoma is the most common subtype (*n* = 551, 1.1%), followed by subtypes of fibrolamellar (*n* = 241, 0.5%), scirrhous (*n* = 82, 0.2%), spindle cell (*n* = 61, 0.1%), and pleomorphic (*n* = 17, ~0%). The patients with fibrolamellar carcinoma were younger and more likely to have a non-cirrhotic liver and better prognoses. Scirrhous carcinoma shared almost the same macro-clinical characteristics and outcomes as the classic HCC. Clear cell carcinoma tended to occur in the Asia-Pacific elderly male population, and more than half of them were large HCC (Size>5cm). Sarcomatoid (including spindle cell and pleomorphic) carcinoma was associated with a larger tumor size, poorer differentiation, and more dismal prognoses. The pathological subtype, T stage, M stage, surgery, alpha-fetoprotein, and cancer history were confirmed as the independent predictors in patients with rare subtypes. The nomogram showed good calibration, discrimination, and net benefits in clinical practice.

**Conclusion:**

The rare subtypes had unique clinicopathological features and biological behaviors compared with the classic HCC. Our findings could provide a valuable reference for clinicians. The constructed nomogram could predict the prognoses with good performance, which is meaningful to individualized management.

## Introduction

Hepatocellular carcinoma (HCC) is an essential component of primary liver cancer. In the last decades, thanks to better etiological monitoring and management, especially the progress of viral hepatitis prevention and therapy, the morbidity of HCC has shown a trend of decelerated growth and is even gradually decreasing both in the East and West. Nevertheless, HCC is still a heavy health burden worldwide (1–7). According to a national survey in the USA, HCC ranks 5th and 7th in estimated new cancer deaths for male and female patients in 2022, respectively (1). In China, HCC had a leading contribution (about 7.9%) to the cancer disability-adjusted life years, second only to lung cancer and gastrointestinal cancer (2). Therefore, a lot of work still needs to be done to improve the HCC patients’ prognoses.

Because of cytological variants, HCC is a highly heterogeneous tumor with several pathological subtypes, mainly including fibrolamellar, scirrhous, clear cell, and sarcomatoid (spindle cell and pleomorphic) carcinoma. Given their unique histologic and clinical characteristics, these subtypes are emerging as distinct entities from classic HCC [HCC, not other specified (NOS)]. However, due to the rarity of atypical subtypes, most existing data primarily stemmed from case reports or single-center cohorts, which lacked sufficient power for conclusive findings to be drawn. Under these circumstances, the primary aim of this study was to compare the epidemiological and clinical features of five rare HCC subtypes (fibrolamellar, scirrhous, clear cell, spindle cell, and pleomorphic), including overall and gender-specific morbidity and incidence-based mortality (IBM), demographics, clinicopathological features, and outcomes, with those of classic HCC utilizing a representative population-based database.

The American Joint Committee on Cancer (AJCC) classifies classic HCC and other rare subtypes under the same category in the 8th edition staging system, which could be debatable—for example, as one of the most well-studied subtypes, patients with fibrolamellar carcinoma are reported to be younger and widely considered to have better prognoses than those with classic HCC (8–13). In this context, categorizing HCC into different subtypes may play a positive role in the individualized management of patients. Meanwhile, considering the wide age distribution and long-term follow-up, some events, the so-called competing risks, such as comorbidities and accidents, may either hinder the observation or modify the occurrence chance of events of interest, which would limit the application of the Kaplan–Meier method and standard Cox regression algorithm in our cohort (14). Therefore, the secondary objective of this study was to conduct competing risk survival analyses and then develop a novel nomogram to evaluate the prognoses of patients with the above-mentioned rare HCC subtypes.

## Patients and Methods

### Patients

**Figure 1:**
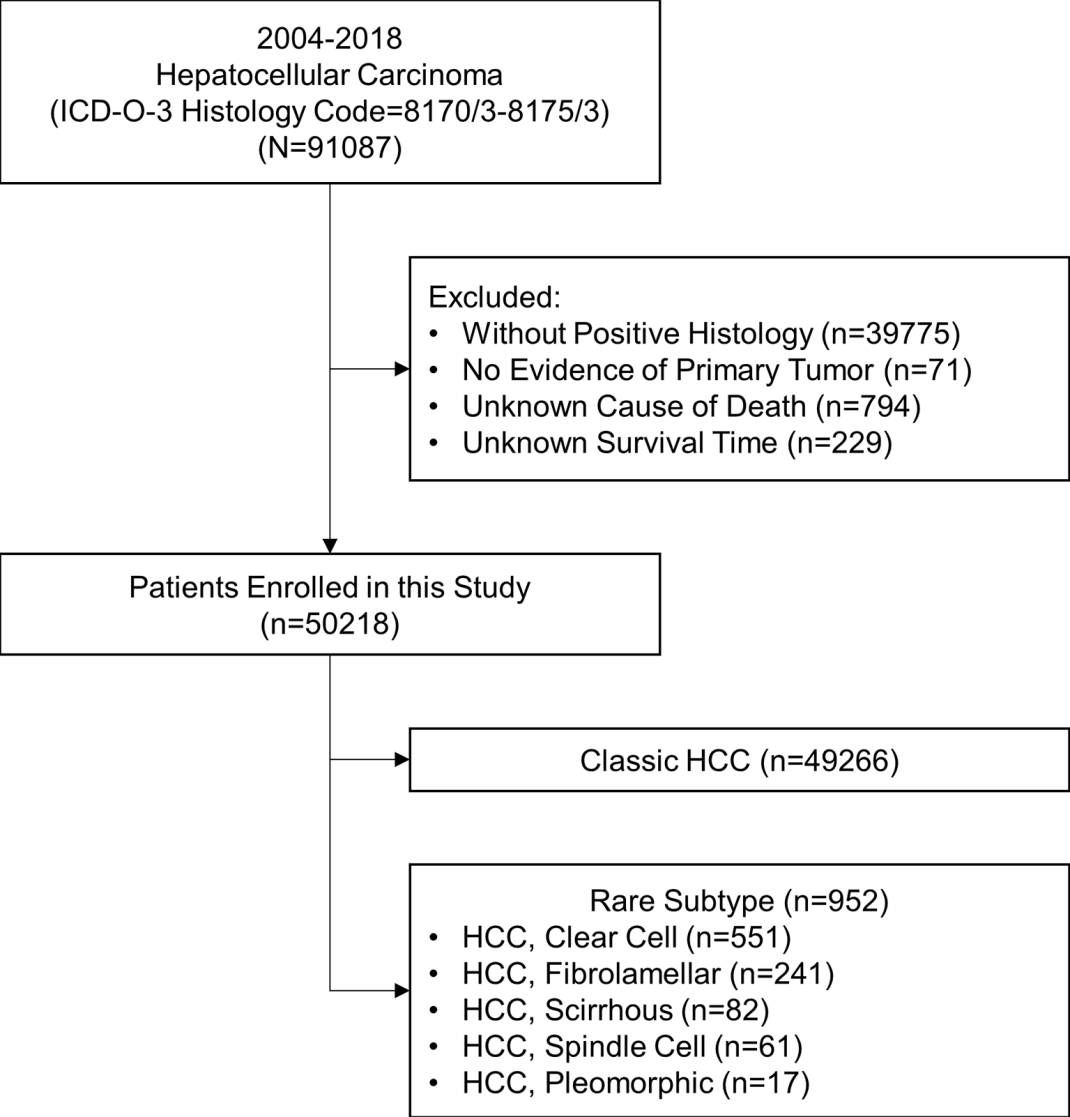
Stepwise extraction process from the Surveillance, Epidemiology, and End Results database. ICD, International Classification of Diseases; HCC, hepatocellular carcinoma.

This study is a retrospective cohort study. Patients diagnosed with different pathological subtypes of HCC (ICD-O-3 Histology Code=8170/3-8175/3) from 2004 to 2018 were extracted from the Surveillance, Epidemiology, and End Results (SEER) research database (18 registries). The SEER database is an authoritative source for cancer statistics and covers about 40.8% of the population in the USA. The data was downloaded with SEER*Stat software (version 8.3.9; The SEER Program, https://seer.cancer.gov). The inclusion criteria were shown as follows: (1) being diagnosed as HCC with positive histology, (2) having evidence of primary tumor, and (3) having a known cause of death and survival time. The stepwise extraction process from the SEER database is shown in **Figure 1**. This study followed the Declaration of Helsinki (as revised in 2013). The SEER database is a public database without personal identifying information. Therefore, the ethical review was exempted, and no consent was needed.

### Definitions

Annual percentage change (APC) was utilized to describe trends of rate. Morbidity and IBM were age-adjusted to the 2000 US standard population. The demographic and clinical factors of patients were obtained from the SEER database. Continuous variables were converted to categorical variables according to the quartile method (age) or the well-accepted cutoff values (tumor size). Cancer-specific survival probability (CSS) and cumulative incidence (CI) of cancer-specific death (CSD) were set as the primary outcomes. All patients were restaged to the current AJCC staging system (8th edition) according to the related fields in the SEER database. The identification of lymph node metastasis (LNM) was strictly based on pathological confirmation, and patients without histologic evidence would be marked as “NX”. The clinical stage referred to the comprehensive AJCC staging, consolidating stages IA and IB into stage I. Missing data and correlations among prognostic factors were considered in survival analyses.

### Statistics

With the hypothesis that the morbidity and IBM changed at a constant percentage from the previous year, the curves were fitted using the Joinpoint Regression Program (version 4.9.0; IMS; Calverton, MD, USA) (15). Survival analyses were performed by univariate and multivariate competing risk models. The cumulative incidences of CSD and other cause-specific death (OCSD) were estimated using the cumulative incidence function (CIF) curves. Propensity score matching (PSM) was used to reduce selection bias between groups. A one-to-one match was performed by the nearest-neighbor method within 0.20 standard deviations between the two groups. Categorical variables were shown as numbers and compared using chi-square test or Fisher’s exact test or likelihood ratio test based on applicable conditions.

The study patients were randomly divided into the training and validation sets with a ratio of 1:1 for external validation. A nomogram was constructed based on independent prognostic factors identified by multivariate competing risk survival analyses to provide a visual tool for clinical use. Harrell’s C-index, Akaike information criterion (AIC), Bayesian information criterion (BIC), and area under receiver operating curve (AUC) were calculated to compare the prognostic performances of the constructed nomogram and the current AJCC staging system. Calibration curves, to evaluate the predictive accuracy of the models, were plotted *via* bootstrapping with 1,000 resamples. Decision curve analysis (DCA), to estimate the clinical utility of the models, was performed by quantifying the net benefits at different threshold probabilities (16). A result was considered statistically significant when two-tailed (*P* < 0.05). All statistical analyses were completed using R software (version 3.6.3; the R Foundation for Statistical Computing, http://www.r-project.org) and SPSS (version 26.0, IBM, Chicago, IL, USA).

## Results

### Morbidity and Incidence-Based Mortality Trends of Different Subtypes of Hepatocellular Carcinoma

**Figure 2:**
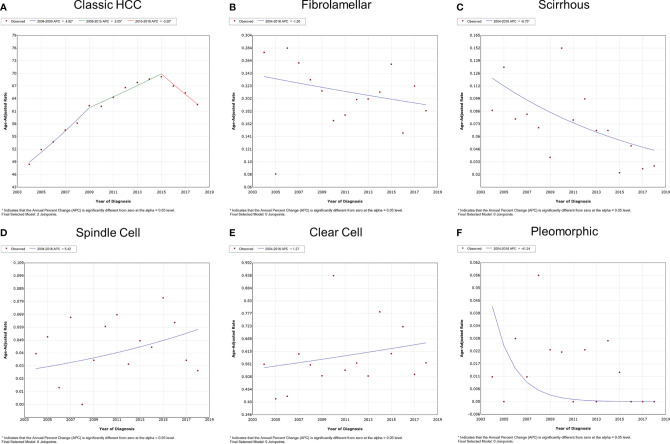
Variation trends for the overall morbidity of different pathological subtypes of HCC from 2004 to 2018. **(A)** Classic HCC, **(B)** fibrolamellar carcinoma, **(C)** scirrhous carcinoma, **(D)** spindle cell carcinoma, **(E)** clear cell carcinoma, and **(F)** pleomorphic carcinoma. HCC, hepatocellular carcinoma.

The overall and gender-specific morbidity trends are displayed in **Figure 2** and **Supplementary Figure S1**. The overall morbidity of subtypes of classic HCC, fibrolamellar, scirrhous, spindle cell, clear cell, and pleomorphic was 48.429, 0.277, 0.087, 0.039, 0.389, 0.011 (per 1,000,000 individuals) in 2004 and 62.607, 0.183, 0.029, 0.026, 0.414, 0 (per 1,000,000 individuals) in 2018, respectively. The morbidity of classic HCC had gone through a process of first rising and then falling, with the APC of 4.8% [95% confidence interval (CI) = 3.9–5.7%, *P* < 0.001] between 2004 and 2009, 2.1% (95% CI = 1.3–2.8%, *P* < 0.001) between 2009 and 2015, and -3.5% (95% CI = -5.0–2.0%, *P* = 0.001) between 2015 and 2018. Meanwhile, the scirrhous variant had a downward morbidity trend from 2004 to 2018, and the APC was -6.8% (95% CI = -12.1–1.0%, *P* = 0.025). The morbidity of other subtypes of HCC remained stable during the period (*P* > 0.05). Similar trends were also observed in both male and female patients. The subgroup analyses also indicated that all subtypes of HCC were apparently dominated by male patients.

**Figure 3:**
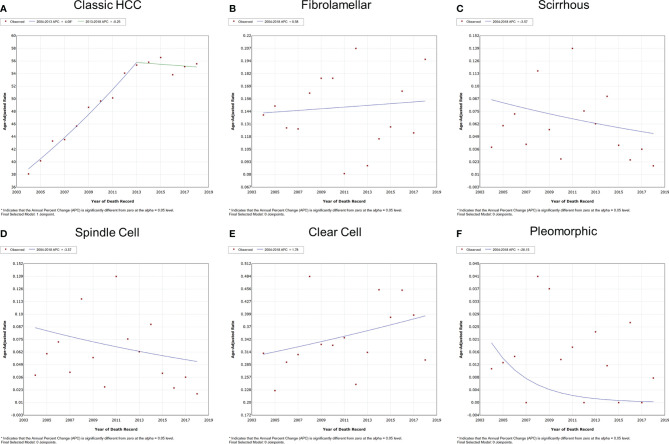
Variation trends for the overall IBM of different pathological subtypes of HCC from 2004 to 2018. **(A)** Classic HCC, **(B)** fibrolamellar carcinoma, **(C)** scirrhous carcinoma, **(D)** spindle cell carcinoma, **(E)** clear cell carcinoma, and **(F)** pleomorphic carcinoma. IBM, incidence-based mortality; HCC, hepatocellular carcinoma.

The overall and gender-specific IBM trends are summarized in **Figure 3** and **Supplementary Figure S2**. The overall IBM of classic HCC had entered a plateau after rising, with the APC of 4.1% (95% CI = 3.5–4.7%, *P* < 0.001) between 2004 and 2013 and -0.3% (95% CI = -1.5–1.0%, *P* = 0.553) between 2013 and 2018. No significant change was observed in the IBM of other subtypes of HCC between 2004 and 2018 (*P* > 0.05). Subgroup analyses also supported these findings according to different genders.

### Baseline Characteristics of Different Subtypes of Hepatocellular Carcinoma

The baseline characteristics are shown in **Table 1** and **Supplementary Table S1**. A total of 50,218 patients were enrolled in this study. The majority of patients (*n* = 49,266, 98.1%) suffered classic HCC, compared to variants where clear cell was the most common pathological subtype (*n* = 551, 1.1%), followed by subtypes of fibrolamellar (*n* = 241, 0.5%), scirrhous (*n* = 82, 0.2%), spindle cell (*n* = 61, 0.1%), and pleomorphic (*n* = 17, ~0%). In terms of age distribution, the fibrolamellar subtype preferred the youth [median: 25, interquartile range (IQR): 17–51 years], whereas the clear cell subtype seemed more likely to occur in older individuals than classic HCC (median: 67, IQR: 59–76 years), which might also cause differences in marital status, cancer history, and OCSD between groups (*P* < 0.05). Although patients who suffered all subtypes of HCC are predominantly male, the relative proportion of females in most rare subtypes was higher than that of classic HCC.

**Table 1:** Baseline characteristics of patients with different pathological subtypes of HCC.

Factors	Classic HCC	Fibrolamellar	Scirrhous	Spindle cell	Clear cell	Pleomorphic
	(*n* = 49,266)	(*n* = 241)	(*n* = 82)	(*n* = 61)	(*n* = 551)	(*n* = 17)
Year of diagnosis			*			
2004–2008	14,939 (30.3)	86 (35.7)	36 (43.9)	11 (18.0)	152 (27.6)	8 (47.1)
2009–2013	16,943 (34.4)	73 (30.3)	29 (35.4)	23 (37.7)	188 (34.1)	6 (35.3)
2014–2018	17,384 (35.3)	82 (34.0)	17 (20.7)	27 (44.3)	211 (38.3)	3 (17.6)
Age		*			*	
≤57	13,123 (26.6)	196 (81.3)	27 (32.9)	16 (26.2)	110 (20.0)	5 (29.4)
57–64	12,245 (24.9)	15 (6.2)	17 (20.7)	16 (26.2)	112 (20.3)	3 (17.6)
64–73	12,162 (24.7)	16 (6.6)	15 (18.3)	15 (24.6)	163 (29.6)	2 (11.8)
>73	11,736 (23.8)	14 (5.8)	23 (28.0)	14 (23.0)	166 (30.1)	7 (41.2)
Gender		*	*		*	
Female	11,285 (22.9)	97 (40.2)	30 (36.6)	16 (26.2)	203 (36.8)	5 (29.4)
Male	37,981 (77.1)	144 (59.8)	52 (63.4)	45 (73.8)	348 (63.2)	12 (70.6)
Race		*			*	
White	33,811 (68.6)	194 (80.5)	59 (72.0)	43 (70.5)	374 (67.9)	10 (58.8)
Asia-Pacific	7,698 (15.6)	20 (8.3)	7 (8.5)	9 (14.8)	111 (20.1)	3 (17.6)
Black	6,942 (14.1)	25 (10.4)	16 (19.5)	9 (14.8)	57 (10.3)	4 (23.5)
Other	815 (1.7)	2 (0.8)	0 (0)	0 (0)	9 (1.6)	0 (0)
Marital status		*			*	
Married	26,022 (52.8)	69 (28.6)	40 (48.8)	34 (55.7)	332 (60.3)	12 (70.6)
Single	13,647 (27.7)	22 (9.1)	29 (35.4)	15 (24.6)	138 (25.0)	2 (11.8)
Other	9,597 (19.5)	150 (62.2)	13 (15.9)	12 (19.7)	81 (14.7)	3 (17.6)
AFP		*				
Negative	10,246 (20.8)	107 (44.4)	19 (23.2)	13 (21.3)	135 (24.5)	2 (11.8)
Positive	25,238 (51.2)	74 (30.7)	44 (53.7)	32 (52.5)	275 (49.9)	13 (76.5)
Borderline/unknown	13,782 (28.0)	60 (24.9)	19 (23.2)	16 (26.2)	141 (25.6)	2 (11.8)
First malignant		*			*	
Yes	41,677 (84.6)	224 (92.9)	69 (84.1)	54 (88.5)	442 (80.2)	17 (100.0)
No	7,589 (15.4)	17 (7.1)	13 (15.9)	7 (11.5)	109 (19.8)	0 (0)
Primary tumor						
Yes	49,012 (99.5)	239 (99.2)	82 (100.0)	61 (100.0)	548 (99.5)	17 (100.0)
No	254 (0.5)	2 (0.8)	0 (0)	0 (0)	3 (0.5)	0 (0)
Neoadjuvant therapy						
Yes	1,644 (3.3)	13 (5.4)	2 (2.4)	2 (3.3)	11 (2.0)	1 (5.9)
No	47,622 (96.7)	228 (94.6)	80 (97.6)	59 (96.7)	540 (98.0)	16 (94.1)
Tumor number						
Single	33,980 (69.0)	178 (73.9)	55 (67.1)	36 (59.0)	389 (70.6)	9 (52.9)
Multiple	15,286 (31.0)	63 (26.1)	27 (32.9)	25 (41.0)	162 (29.4)	8 (47.1)
		*			*	*
Tumor size	(*n* = 39,537)	(*n* = 211)	(*n* = 71)	(*n* = 52)	(*n* = 457)	(*n* = 16)
≤2 cm	4,579 (11.6)	10 (4.7)	8 (11.3)	5 (9.6)	33 (7.2)	0 (0)
2–5 cm	15,950 (40.3)	30 (14.2)	24 (33.8)	14 (26.9)	152 (33.3)	4 (25.0)
5cm	19,008 (48.1)	171 (81.0)	39 (54.9)	33 (63.5)	272 (59.5)	12 (75.0)
Surgery		*			*	*
None	32,650 (66.3)	99 (41.1)	59 (72.0)	46 (75.4)	330 (59.9)	9 (52.9)
LD	4,938 (10.0)	5 (2.1)	5 (6.1)	4 (6.6)	45 (8.2)	1 (5.9)
LR	7,314 (14.8)	122 (50.6)	12 (14.6)	9 (14.8)	159 (28.9)	7 (41.2)
LT	3,860 (7.8)	13 (5.4)	6 (7.3)	2 (3.3)	14 (2.5)	0 (0)
Method unknown	504 (1.0)	2 (0.8)	0 (0)	0 (0)	3 (0.5)	0 (0)
Radiotherapy						
Yes	4,223 (8.6)	19 (7.9)	8 (9.8)	4 (6.6)	48 (8.7)	1 (5.9)
No/unknown	45,043 (91.4)	222 (92.1)	74 (90.2)	57 (93.4)	503 (91.3)	16 (94.1)
Chemotherapy		*				
Yes	17,104 (34.7)	117 (48.5)	23 (28.0)	15 (24.6)	177 (32.1)	2 (11.8)
No/unknown	32,162 (65.3)	124 (51.5)	59 (72.0)	46 (75.4)	374 (67.9)	15 (88.2)
T stage		*			*	
T1a	3,068 (6.2)	7 (2.9)	5 (6.1)	1 (1.6)	25 (4.5)	0 (0)
T1b	14,669 (29.8)	85 (35.3)	27 (32.9)	9 (14.8)	201 (36.5)	6 (35.3)
T1NOS	2,125 (4.3)	8 (3.3)	4 (4.9)	4 (6.6)	26 (4.7)	0 (0)
T2	8,816 (17.9)	35 (14.5)	16 (19.5)	14 (23.0)	82 (14.9)	4 (23.5)
T3	7,032 (14.3)	42 (17.4)	15 (18.3)	14 (23.0)	86 (15.6)	5 (29.4)
T4	5875 (11.9)	45 (18.7)	8 (9.8)	10 (16.4)	64 (11.6)	1(5.9)
TX	7 681 (15.6)	19 (7.9)	7 (8.5)	9 (14.8)	67 (12.2)	1 (5.9)
N stage		*	*			
N0	2 147 (4.4)	39 (16.2)	9 (11.0)	5 (8.2)	28 (5.1)	1 (5.9)
N1	107 (0.2)	41 (17.0)	0 (0)	0 (0)	2 (0.4)	0 (0)
NX	47 011 (95.4)	161 (66.8)	73 (89.0)	56 (91.8)	521 (94.6)	16 (94.1)
M stage		*		*		
M0	40 871 (83.0)	178 (73.9)	67 (81.7)	35 (57.4)	468 (84.9)	17 (100.0)
M1	8 395 (17.0)	63 (26.1)	15 (18.3)	26 (42.6)	83 (15.1)	0 (0)
		*		*	*	*
Grade	(*n* = 29,149)	(*n* = 107)	(*n* = 52)	(*n* = 33)	(*n* = 334)	(*n* = 14)
G1	9,194 (31.5)	22 (20.6)	12 (23.1)	1 (3.0)	78 (23.4)	1 (7.1)
G2	13,176 (45.2)	61 (57.0)	25 (48.1)	3 (9.1)	181 (54.2)	0 (0)
G3–G4	6,779 (23.3)	24 (22.4)	15 (28.8)	29 (87.9)	75 (22.5)	13 (92.9)
		*			*	
Ishak score	(*n* = 11,156)	(*n* = 41)	(*n* = 17)	(*n* = 12)	(*n* = 137)	(*n* = 4)
0–4	3,135 (28.1)	37 (90.2)	4 (23.5)	6 (50.0)	69 (50.4)	2 (50.0)
5–6	8,021 (71.9)	4 (9.8)	13 (76.5)	6 (50.0)	68 (49.6)	2 (50.0)
CI of CSD		*		*		*
1 year	0.450	0.236	0.452	0.707	0.401	0.588
3 year	0.614	0.460	0.615	0.827	0.585	0.824
5 year	0.671	0.551	0.697	0.827	0.655	–
CI of OCSD		*				
1 year	0.065	<0.001	0.062	0.069	0.064	0.059
3 year	0.102	0.019	0.077	0.069	0.105	0.118
5 year	0.120	0.025	0.093	0.069	0.116	–

HCC, hepatocellular carcinoma; AFP, alpha-fetoprotein; LD, local destruction; LR, liver resection; LT, liver transplantation; CI, cumulative incidence; CSD, cancer-specific death; OCSD, other cause-specific death.

*P < 0.05 when compared with classic HCC. (The P-values are shown in **Supplementary Table S1**).

G1, well differentiated; G2, moderately differentiated; G3–G4, poorly differentiated/undifferentiated.

In all cases with recorded tumor size, fibrolamellar (81.0%), spindle cell (63.5%), clear cell (59.5%), and pleomorphic (75.0%) subtypes were more frequent than classic HCC (48.1%) to present large liver cancer (>5 cm), with a median size of 10.0 (IQR: 6.4–14.0) cm, 8.0 (IQR: 3.9–11.4) cm, 6.2 (IQR: 4.0–9.6) cm, and 10.8 (IQR: 9.8–13.0) cm, respectively (*P* < 0.05). The tumor size also limited the decision-making of surgical approaches to a large extent, especially the choice of liver resection (LR) and liver transplantation (LT) (*P* < 0.05). More than one-third of fibrolamellar (36.1%), spindle cell (39.4%), and pleomorphic (35.3%) cases suffered locally advanced tumors (T3–T4). Sarcomatoid HCC, including spindle cell (87.9%) and pleomorphic (92.9%) subtypes, was mostly poorly differentiated or undifferentiated (G3–G4). Compared with classic HCC (71.9%), liver cirrhosis was less common in patients with fibrolamellar (9.8%) and clear cell (49.6%) subtypes (*P* < 0.05). Fibrolamellar cases had a significantly higher lymph node dissection (LND) rate (33.2%) than other subtypes (4.6–11.0%). LNM was observed in patients suffering from classic HCC, fibrolamellar subtype, and clear cell subtype, with the incidence of 4.7% (107/2,254), 51.2% (41/80), and 6.7% (2/30), respectively.

**Figure 4:**
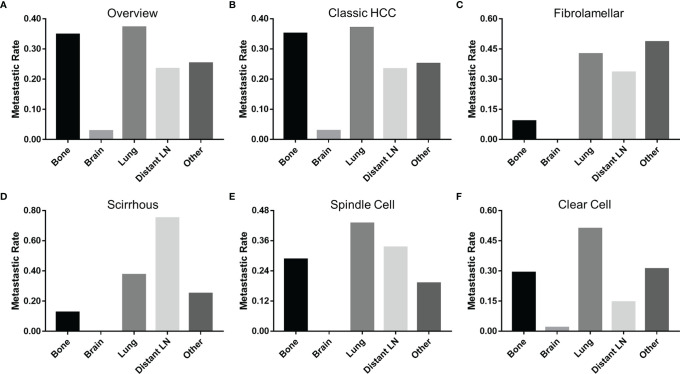
Incidence of extrahepatic distant metastases at different sites of HCC. **(A)** All pathological subtypes, **(B)** classic HCC, **(C)** fibrolamellar carcinoma, **(D)** scirrhous carcinoma, **(E)** spindle carcinoma, and **(F)** clear cell carcinoma. HCC, hepatocellular carcinoma; LN, lymph node.

Patients with fibrolamellar (26.1%) or spindle cell (42.6%) subtypes were more prone to distant metastasis (M1) than those with classic HCC (17.0%, *P* < 0.05). Because the data on metastatic sites was incomplete before 2010, a total of 5,460 M1 patients (5,343 patients with classic HCC, 33 patients with fibrolamellar carcinoma, 8 patients with scirrhous carcinoma, 21 patients with spindle cell carcinoma, and 55 patients with clear cell carcinoma) were included for subsequent analyses. As shown in **Figure 4**, the lung was the most vulnerable site of extrahepatic distant metastases in patients with classic HCC, spindle cell, and clear cell subtypes, followed by bone (classic HCC and clear cell) or distant lymph node (spindle cell). However, the same results were not obtained in those who had fibrolamellar or scirrhous subtypes.

### Prognoses of Different Subtypes of Hepatocellular Carcinoma

**Figure 5:**
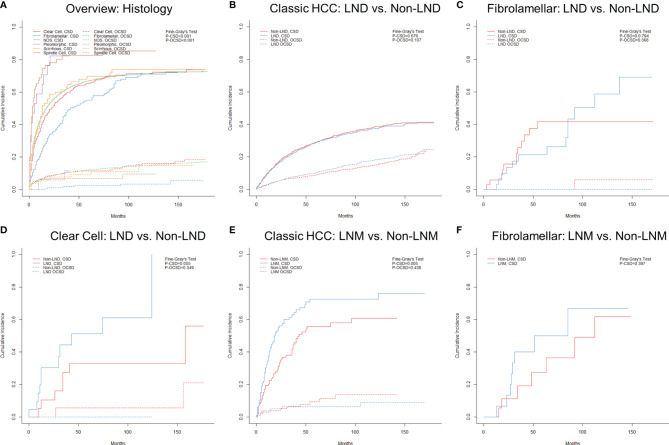
Cumulative incidence function curves of mortality in hepatocellular carcinoma patients stratified by different factors: **(A)** histology and **(B–F)** nodal status. LND, lymph node dissection; LNM, lymph node metastasis; CSD, cancer-specific death; OCSD, other cause-specific death.

The final follow-up was performed in November 2020, with a mean follow-up time of 23.8 ± 33.8 (IQR: 2–30) months. During the follow-up period, 37,955 (75.6%) patients died, and 84.0% of deaths were attributable to various subtypes of HCC. The median cancer-specific survival time of subtypes of classic HCC, fibrolamellar, scirrhous, spindle cell, clear cell, and pleomorphic was 15.0 (95% CI = 14.6–15.4), 41.0 (95% CI = 29.0–53.0), 13.0 (95% CI = 8.5–17.5), 3.0 (95% CI = 1.0–5.0), 19.0 (95% CI = 14.7–23.3), and 5.0 (95% CI = 0–11.7) months, respectively. The cumulative incidence of CSD and OCSD is displayed in **Table 1** and **Figure 5A**. The fibrolamellar carcinoma had the best cancer-specific survival (*P* < 0.05). Classic HCC, scirrhous, and clear cell subtypes shared similar outcomes (*P* > 0.05). However, patients with sarcomatoid HCC (spindle and pleomorphic subtypes) would suffer dismal prognoses (*P* < 0.05).

As displayed in **Supplementary Figure S3**, the clinical stage (or AJCC comprehensive stage) did not appear to show a good discrimination among rare subtypes, especially stage IVA (N1M0). The effect of nodal status on prognoses was further analyzed in subtypes with a sufficient sample size. For patients with classic HCC, the LND group did not show better prognoses than the non-LND group (**Supplementary Table S2** and **Figure 5B**; *P* = 0.676) after PSM, which also applied to those with fibrolamellar or clear cell subtypes (**Supplementary Tables S3**, **S4**, and **Figures 5C, D**; *P* > 0.05). Unsurprisingly, classic HCC patients with LNM were confirmed to have worse chances of survival (**Supplementary Table S5** and **Figure 5E**; *P* = 0.005). However, there was no survival difference between LNM and non-LNM in patients with fibrolamellar subtype after PSM (**Supplementary Table S6** and **Figure 5F**; *P* = 0.397).

The CIF curves stratified by different surgical approaches are shown in **Supplementary Figure S4**. Overall, patients receiving liver-directed therapy (local destruction, LR, and LT) would have better prognoses. A truly matched study to compare the value of LR and LT was not possible with the limited number of cases, so we summarized the clinical characteristics of LT recipients in the form of a case series (**Supplementary Table S7**).

### Development and Validation of a Model for Predicting the Prognoses of Rare Subtypes of Hepatocellular Carcinoma

Further survival analyses were performed in 952 patients with rare subtypes other than classic HCC. The study patients were randomly divided into the training set (*n* = 476) and the validation set (*n* = 476) with a ratio of 1:1. The baseline characteristics of the training and validation sets are summarized in **Supplementary Table S8**. As shown in **Supplementary Tables S9**, **S10**, and **Supplementary Figure S5**, multivariate competing risk analyses finally confirmed the pathological subtype, T stage, M stage, surgery, alpha-fetoprotein (AFP), and cancer history as the independent prognostic factors of CSD in the training set (*P* < 0.05).

**Figure 6:**
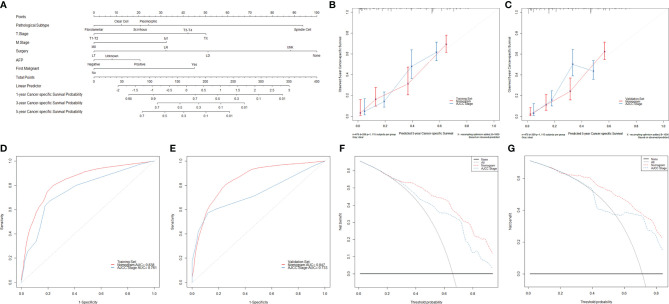
Developing and validating a novel model to predict the prognoses of patients with rare pathological subtypes of hepatocellular carcinoma. **(A)** The nomogram to predict cancer-specific survival was developed from the training set. **(B, C)** Calibration curve analyses of the nomogram and the current AJCC staging system (8th edition) to evaluate the prognosis effects at the 5-year point in the training and validation sets. **(D, E)** Receiver operating characteristic curve analyses of the nomogram and the current AJCC staging system (8th edition) to evaluate the prognosis effects at the 5-year point in the training and validation sets. **(F, G)** Decision curve analyses of the nomogram and the current AJCC staging system (8th edition) to evaluate the prognosis effects at the 5-year point in the training and validation sets. LT, liver transplantation; LR, liver resection; LD, local destruction; UNK, unknown; AFP, alpha-fetoprotein.

The nomogram to predict the CSS of rare subtypes of HCC was developed based on the independent prognostic factors (**Figure 6A**), with C-index of 0.776 (95% CI = 0.739–0.812) in the training set and 0.749 (95% CI = 0.723–0.775) in the validation set. The calibration curves showed good consistency between the predicted and the observed CSS in both the training and validation sets (**Figures 6B, C** and **Supplementary Figures S6A–D**). As shown in **Figures 6D, E** and **Supplementary Figures S6E–H**, the constructed nomogram’s 1-, 3-, and 5-year receiver operating characteristic values were 0.813, 0.831, and 0.838 in the training set and 0.781, 0.838, and 0.847 in the validation set. Compared with the current AJCC staging system (8th edition), the nomogram had lower AIC and BIC values in the training and validation set, indicating a better discriminative capacity (**Table 2**). To further estimate the clinical utility of the two models, DCAs were plotted in **Figures 6F, G** and **Supplementary Figures S6I–L**. The nomogram provided a better net clinical benefit than “treat all” or “treat none” schemes and the current AJCC staging system. To further simplify the application of the nomogram, an online tool has been produced and published, which can be accessed through the following URL: https://chenxiaoyuan.shinyapps.io/RareHCC/.

**Table 2:** Comparison of prognostic performances between the constructed nomogram and the AJCC staging system.

Models	C-index (95% CI)	AIC	BIC	1-year AUC	3-year AUC	5-year AUC
Training set (*n* = 476)						
Nomogram	0.776 (0.739–0.812)	3,191.681	3, 242.963	0.813	0.831	0.838
AJCC stage	0.689 (0.647–0.731)	3, 272.167	3, 305.133	0.739	0.757	0.761
Validation set (*n* = 476)						
Nomogram	0.749 (0.723–0.775)	3,180.161	3,183.894	0.781	0.838	0.847
AJCC stage	0.657 (0.626–0.689)	3, 274.712	3, 278.446	0.662	0.720	0.733

AJCC, American Joint Committee on Cancer; CI, confidence interval; AIC, Akaike information criterion; BIC, Bayesian information criterion; AUC, area under the curve.

## Discussion

In the era of precision medicine, high heterogeneity poses great challenges in the prevention, diagnosis, and treatment of HCC. Due to the rarity of atypical HCC subtypes, it is really hard for a single institute to accumulate enough cases for in-depth research. In the present study, we recruited a high-volume cohort of 50,218 patients from the SEER database to macroscopically describe the epidemiological and clinical spectrum of different HCC pathological subtypes. Furthermore, we developed and validated an online nomogram based on the competing risk model to predict the prognoses of patients suffering from rare subtypes of HCC, which could provide more evidence for individualized management.

Between 2004 and 2018, the morbidity of classic HCC began to show a downward trend. Two possible reasons might explain this change: first, the increased availability and penetration of antiviral therapy slowed the progression of “hepatitis–cirrhosis–liver cancer” (especially hepatitis B in the East and hepatitis C in the West); second, as another emerging carcinogenic factor, nonalcoholic fatty liver disease (NAFLD) and metabolic syndrome have rapidly rising morbidity and population attributable fraction. Considering that the progression from NAFLD to liver cancer is a long-term process, it seemed still that the incidence peak of NAFLD-related liver cancer has not yet arrived (1, 2, 6, 17, 18). In addition to scirrhous carcinoma, the morbidity of other rare subtypes remained stable during this period. Currently, no specific exposure factor for these rare subtypes has been identified. Meanwhile, we observed differences in demographics and cirrhosis rate between classic HCC and rare subtypes in our cohort, indicating that rare HCC subtypes need to be better understood at the etiological level. As for IBM, all subtypes in this study were plateau, that is, IBM showed no further increase or significant decrease. Therefore, more attention should still be paid to developing better therapeutic strategies to further improve the prognoses of HCC patients.

In general, classic HCC is characterized by a lack of fibrous stroma, but there exist two rare subtypes of fibrolamellar and scirrhous carcinoma with abundant fibrous stroma (19–25). As the second common rare subtype in our cohort, fibrolamellar carcinoma was first described by Edmondson in 1956 (26) and named by Craig in 1980 (27). Compared with classic HCC, patients with fibrolamellar carcinoma were observed to be younger, have a greater proportion of Caucasians and female patients, and be less likely to have positive AFP and liver cirrhosis, which was consistent with previous studies (8, 10–12, 28). These findings also explained the differences in some other variables, such as marital status, cancer history, and OCSD. Meanwhile, similar to intrahepatic cholangiocarcinoma and combined hepatocellular–cholangiocarcinoma, fibrolamellar carcinoma tended to have a larger tumor size and higher probability of extrahepatic invasion (lymph node and distant metastasis) (29, 30). However, these factors seemed not to be drivers of poor prognoses as expected. Younger age and better liver reserve function allowed patients with fibrolamellar carcinoma to tolerate LR well. For this reason, the majority (89.2%, 122/142) of enrolled patients received LR, and over half (54.9%, 67/122, data not shown) of them were major resection. Atienza et al. (11) reported the promising role of LT in patients with fibrolamellar carcinoma with 1-, 3-, and 5-year overall survival of 96%, 80%, and 48%. In this study, the 1-, 3-, and 5-year CSD of LT recipients were 0, 30.8%, 46.2%, similar to the previous study and the LR group (5.8%, 21.8%, and 31.3%). Notably, Atienza and colleagues did not directly compare the therapeutic value of LT and LR, and no further PSM analysis was also conducted in our cohort due to the limited sample size. Considering the scarcity of donor livers, the role of LT in patients with fibrolamellar carcinoma still needs to be viewed with caution.

As another HCC subtype rich in stromal components, scirrhous carcinoma shared almost the same macroscopical clinical characteristics and prognoses with classic HCC, which was also supported by other researchers (19, 21, 31–33). Interestingly, both the two rare subtypes might present unusual paraneoplastic manifestations: hyperammonemic encephalopathy (HAE) occurred in patients with fibrolamellar carcinoma, and hypercalcemia was found in scirrhous carcinoma sufferers (34–37). However, for most patients, vague symptoms and nonspecific imaging features presented challenges in differential diagnoses of scirrhous carcinoma, especially from fibrolamellar carcinoma and cholangiocarcinoma. Unlike fibrolamellar, whose stroma is composed of dense lamellated collagenous bands with sparse cellular components, the stroma of scirrhous carcinoma is more complex, showing more abundant cancer-associated fibroblasts, tumor-infiltrating macrophages, and stemness-related marker expression (22, 24, 38). A team from Heidelberg University summarized the immunohistochemical (IHC) features of scirrhous carcinoma, and the positive rate of HepPar-1, CK7, CK19 and EMA was 64.6%, 40.7%, 16.0%, and 41.9%, respectively (19). Among these markers, CK7 and CK19 could be used to differentiate from classic HCC, and HepPar-1 could be used to distinguish from cholangiocarcinoma. It was reported that CD68 had a high positive rate in fibrolamellar carcinoma but was usually negative in scirrhous carcinoma, suggesting another strong differential marker (22, 24, 38–40). Nevertheless, these markers are still nonspecific, and gene-level detection could provide new ideas for diagnosis. Different from TP53 and CTNNB1 mutations in classic HCC, fibrolamellar carcinoma frequently harbors DNAJB1-PRKACA fusion transcript (except for patients with Carney syndrome), and scirrhous carcinoma was reported to be associated with TSC1/TSC2 mutations (39, 41, 42).

Clear cell carcinoma is defined to consist of mixtures of clear and/or acidophilic ground glass hepatocytes with excessive glycogen and/or fat (43). In our cohort, clear cell carcinoma tended to occur in the Asia-Pacific elderly male population with both cirrhotic and non-cirrhotic livers, and more than half of them were large HCC (Size>5cm). LR was still the most mainstream surgical approach (71.9%) with a 5-year CSD of 44.1%. A total of 14 patients received LT during the same period, and the 5-year CSD was 14.9%, indicating a potential therapeutic value of LT but needs to be further confirmed. Contradicting with two previous studies based on the national cancer database (NCDB), clear cell carcinoma was the most common rare HCC subtype in this study (10, 12). In addition to differences in the data source, another possible reason is the ill-defined pathologically diagnostic criteria: some researchers consider that 30% of clear cells is sufficient, but others diagnose clear cell carcinoma only when the tumors contain no less than 50% of clear cells (44–47). Unfortunately, data regarding clear cell components were not subgroup analyzed in our cohort due to unavailable data in the SEER database, which was also identified as an independent prognostic factor in some prior reports (46, 47). However, just as Xu et al. pointed out, it is hard to accurately account for the proportion of clear cells in tumors due to various presence patterns (46). Considering the finding obtained from single-center studies with small sample sizes, the prognostic role of clear cell components still deserves to be debated in subsequent studies.

Sarcomatoid carcinoma is a highly complicated subtype consisting of spindle and pleomorphic cells with or without multinucleated giant cells (48). Currently, little is known about the occurrence and development of sarcomatoid carcinoma. Several hypotheses have been proposed, such as epithelial-mesenchymal transition (EMT), inflammatory responses, stochastic phenotype switching, pluripotent precursor cell or stem cell hypothesis, and mutations in Gene CDKN2A (48–53). In our cohort, sarcomatoid carcinoma was observed to be significantly associated with larger tumor size, poorer differentiation, and much more dismal prognoses. The therapy strategy remains fragmented for this vulnerable patient population. Systematic therapy has been reported to have a limited effect in several case reports (54–57). For study patients with active liver-directed therapy, the 3-year CSD was as high as 63.3% of the spindle subtype and 62.5% of the pleomorphic subtype, respectively (data not shown; the 5-year CSD could not be calculated). These pessimistic findings were also confirmed by other studies (49, 58–60).

Subsequent competing risk survival analyses were conducted to develop a novel model better to evaluate the prognoses of patients with rare HCC subtypes. The pathological subtype, T stage, M stage, surgery, AFP, and cancer history were confirmed as the independent prognostic factors for cancer-specific survival in the multivariable analyses, and then a nomogram was developed based on these variables. In addition, we also provided a companion online tool for individualized evaluation. The nomogram showed high accuracy with C-indexes exceeding 0.700 and well-fitted calibration curves in both the training and validation sets. Besides these, the nomogram displayed better goodness of fit according to its lower AIC and BIC values. The DCA also confirmed the validity of the nomogram for prediction and demonstrated that the nomogram had better clinical value than the current staging system.

As far as we know, this study is the largest cohort focusing on the cancer-specific survival of rare HCC pathological subtypes and the first one to provide a competing risk analysis-based model for prognoses evaluation. Although our study has many merits, including but not limited to representative data sources, high-volume sample size, and complete long-term follow-up, some limitations still exist. First, the major drawback is the study design of the retrospective study, which could introduce inherent bias. Second, the SEER database lacks detailed clinicopathological data, including the Child–Pugh grade, adjuvant therapy regimens, and microscopic or IHC features, making it difficult to perform further PSM or subgroup analyses. Third, the SEER database does not provide data on pathological sampling methods. Different sampling methods may create barriers for differential diagnosis among different subtypes of HCC, such as needle biopsy and surgical resection, which may lead to a potential bias. Fourth, we could not model each subtype separately due to the limited sample size. In this study, we treated all rare subtypes as a whole and set “pathological subtype” as a variable in survival analyses. Despite the seemingly reduced accuracy, the constructed nomogram achieved better predictions than the current AJCC staging system and could benefit more patients. Under these circumstances, we believe the sacrifice is worthwhile.

## Conclusion

Rare pathological subtypes have highly heterogeneous epidemiological and clinical features from classic HCC. Fibrolamellar carcinoma has the best prognosis, while the spindle cell and pleomorphic subtypes have the worst. The pathological subtype, T stage, M stage, surgery, AFP, and cancer history were confirmed as the independent prognostic factors for cancer-specific survival in patients with rare HCC subtypes. The constructed nomogram is well validated and could predict the prognoses with good performance, which is meaningful to individualized management.

## Data Availability Statement

Publicly available datasets were analyzed in this study. This data can be found here: https://seer.cancer.gov.

## Ethics Statement

Ethical review and approval was not required for the study on human participants in accordance with the local legislation and institutional requirements. Written informed consent from the participants’ legal guardian/next-of-kin was not required to participate in this study in accordance with the national legislation and the institutional requirements.

## Author Contributions

XW is the lead contact for this article. XW, YG, and XC conceived and designed the study. XW and YG supervised the study and offered administrative support. YL, XS, LZ, and JZ collected and assembled the data. XC, YL, GH, and CN analyzed and interpreted the data. XC and XS wrote the manuscript. XW and YG reviewed the manuscript. All authors contributed to the article and approved the submitted version.

## Funding

This study was supported by grants from the National Natural Science Foundation of China (grant numbers 31930020, 81870488, 81521004, and 81530048), the Natural Science Foundation of Jiangsu Province (grant number BK20170142), and the Key Laboratory of Liver Transplantation, Chinese Academy of Medical Sciences (grant numbers 2018PT31043 and 2019PT320015).

## Conflict of Interest

The authors declare that the research was conducted in the absence of any commercial or financial relationships that could be construed as a potential conflict of interest.

## Publisher’s Note

All claims expressed in this article are solely those of the authors and do not necessarily represent those of their affiliated organizations, or those of the publisher, the editors and the reviewers. Any product that may be evaluated in this article, or claim that may be made by its manufacturer, is not guaranteed or endorsed by the publisher.

## References

[1] Siegel RL Miller KD Fuchs HE Jemal A. Cancer Statistics, 2022. CA Cancer J Clin (2022) 72(1):7–33. doi: 10.3322/caac.21708 35020204

[2] Qiu H Cao S Xu R. Cancer Incidence, Mortality, and Burden in China: A Time-Trend Analysis and Comparison With the United States and United Kingdom Based on the Global Epidemiological Data Released in 2020. Cancer Commun (Lond) (2021) 41(10):1037–48. doi: 10.1002/cac2.12197 PMC8504144 34288593

[3] Llovet JM Kelley RK Villanueva A Singal AG Pikarsky E Roayaie S. Hepatocellular Carcinoma. Nat Rev Dis Primers (2021) 7(1):6. doi: 10.1038/s41572-020-00240-3 33479224 PMC13537433

[4] Kudo M Izumi N Kokudo N Sakamoto M Shiina S Takayama T. Report of the 22nd Nationwide Follow-Up Survey of Primary Liver Cancer in Japan (2012-2013). Hepatol Res (2022) 52(1):5–66. doi: 10.1111/hepr.13675 34050584

[5] Xia YX Zhang F Li XC Kong LB Zhang H Li DH. Surgical Treatment of Primary Liver Cancer:a Report of 10 966 Cases. Zhonghua Wai Ke Za Zhi (2021) 59(1):6–17. doi: 10.3760/cma.j.cn112139-20201110-00791 33412628

[6] Kulik L El-Serag HB. Epidemiology and Management of Hepatocellular Carcinoma. Gastroenterology (2019) 156(2):477–491.e1. doi: 10.1053/j.gastro.2018.08.065 30367835 PMC6340716

[7] Chen XY Lu YW Shi XL Han GY Zhao J Gao Y. Development and Validation of a Novel Model to Predict Regional Lymph Node Metastasis in Patients With Hepatocellular Carcinoma. Front Oncol (2022) 12:835957. doi: 10.3389/fonc.2022.835957 35223515 PMC8874317

[8] Ramai D Ofosu A Lai JK Gao ZH Adler DG. Fibrolamellar Hepatocellular Carcinoma: A Population-Based Observational Study. Dig Dis Sci (2021) 66(1):308–14. doi: 10.1007/s10620-020-06135-3 32052215

[9] McDonald JD Gupta S Shindorf ML Gamble LA Ruff SM Drake J. Elevated Serum α-Fetoprotein is Associated With Abbreviated Survival for Patients With Fibrolamellar Hepatocellular Carcinoma Who Undergo a Curative Resection. Ann Surg Oncol (2020) 27(6):1900–5. doi: 10.1245/s10434-019-08178-x PMC8456707 31925595

[10] Zakka K Jiang R Alese OB Shaib WL Wu C Wedd JP. Clinical Outcomes of Rare Hepatocellular Carcinoma Variants Compared to Pure Hepatocellular Carcinoma. J Hepatocell Carcinoma (2019) 6:119–29. doi: 10.2147/JHC.S215235 PMC6660638 31413960

[11] Atienza LG Berger J Mei X Shah MB Daily MF Grigorian A. Liver Transplantation for Fibrolamellar Hepatocellular Carcinoma: A National Perspective. J Surg Oncol (2017) 115(3):319–23. doi: 10.1002/jso.24515 27878821

[12] Jernigan PL Wima K Hanseman DJ Hoehn RS Ahmad SA Shah SA. Natural History and Treatment Trends in Hepatocellular Carcinoma Subtypes: Insights From a National Cancer Registry. J Surg Oncol (2015) 112(8):872–6. doi: 10.1002/jso.24083 26603598

[13] Mayo SC Mavros MN Nathan H Cosgrove D Herman JM Kamel I. Treatment and Prognosis of Patients With Fibrolamellar Hepatocellular Carcinoma: A National Perspective. J Am Coll Surg (2014) 218(2):196–205. doi: 10.1016/j.jamcollsurg.2013.10.011 24315886 PMC4596238

[14] Austin PC Fine JP. Practical Recommendations for Reporting Fine-Gray Model Analyses for Competing Risk Data. Stat Med (2017) 36(27):4391–400. doi: 10.1002/sim.7501 PMC5698744 28913837

[15] Kim HJ Fay MP Feuer EJ Midthune DN. Permutation Tests for Joinpoint Regression With Applications to Cancer Rates. Stat Med (2000) 19(3):335–51. doi: 10.1002/(sici)1097-0258(20000215)19:3<335::aid-sim336>3.0.co;2-z 10649300

[16] Vickers AJ Elkin EB. Decision Curve Analysis: A Novel Method for Evaluating Prediction Models. Med Decis Making (2006) 26(6):565–74. doi: 10.1177/0272989X06295361 PMC2577036 17099194

[17] Powell EE Wong VW Rinella M. Non-Alcoholic Fatty Liver Disease. Lancet (2021) 397(10290):2212–24. doi: 10.1016/S0140-6736(20)32511-3 33894145

[18] Huang DQ El-Serag HB Loomba R. Global Epidemiology of NAFLD-Related HCC: Trends, Predictions, Risk Factors and Prevention. Nat Rev Gastroenterol Hepatol (2021) 18(4):223–38. doi: 10.1038/s41575-020-00381-6 PMC8016738 33349658

[19] Murtha-Lemekhova A Fuchs J Schulz E Sterkenburg AS Mayer P Pfeiffenberger J. Scirrhous Hepatocellular Carcinoma: Systematic Review and Pooled Data Analysis of Clinical, Radiological, and Histopathological Features. J Hepatocell Carcinoma (2021) 8:1269–79. doi: 10.2147/JHC.S328198 PMC8547765 34712626

[20] O’Neill AF Church AJ Perez-Atayde AR Shaikh R Marcus KJ Vakili K. Fibrolamellar Carcinoma: An Entity All its Own. Curr Probl Cancer (2021) 45(4):100770. doi: 10.1016/j.currproblcancer.2021.100770 34272087

[21] Huang SC Liao SH Su TH Jeng YM Kao JH. Clinical Manifestations and Outcomes of Patients With Scirrhous Hepatocellular Carcinoma. Hepatol Int (2021) 15(2):472–81. doi: 10.1007/s12072-021-10146-1 33544314

[22] Samdanci ET Akatli AN Soylu NK. Clinicopathological Features of Two Extremely Rare Hepatocellular Carcinoma Variants: A Brief Review of Fibrolamellar and Scirrhous Hepatocellular Carcinoma. J Gastrointest Cancer (2020) 51(4):1187–92. doi: 10.1007/s12029-020-00500-1 32860202

[23] Lin CC Yang HM. Fibrolamellar Carcinoma: A Concise Review. Arch Pathol Lab Med (2018) 142(9):1141–5. doi: 10.5858/arpa.2017-0083-RS 30141990

[24] Kim YJ Rhee H Yoo JE Alves VAF Kim GJ Kim HM. Tumour Epithelial and Stromal Characteristics of Hepatocellular Carcinomas With Abundant Fibrous Stroma: Fibrolamellar Versus Scirrhous Hepatocellular Carcinoma. Histopathology (2017) 71(2):217–26. doi: 10.1111/his.13219 28326574

[25] Kassahun WT. Contemporary Management of Fibrolamellar Hepatocellular Carcinoma: Diagnosis, Treatment, Outcome, Prognostic Factors, and Recent Developments. World J Surg Oncol (2016) 14(1):151. doi: 10.1186/s12957-016-0903-8 27215576 PMC4877801

[26] Edmondson HA. Differential Diagnosis of Tumors and Tumor-Like Lesions of Liver in Infancy and Childhood. AMA J Dis Child (1956) 91(2):168–86. doi: 10.1001/archpedi.1956.02060020170015 13282629

[27] Craig JR Peters RL Edmondson HA Omata M. Fibrolamellar Carcinoma of the Liver: A Tumor of Adolescents and Young Adults With Distinctive Clinico-Pathologic Features. Cancer (1980) 46(2):372–9. doi: 10.1002/1097-0142(19800715)46:2<372::aid-cncr2820460227>3.0.co;2-s 6248194

[28] Takahashi A Imamura H Ito R Kawano F Gyoda Y Ichida H. A Case Report of Fibrolamellar Hepatocellular Carcinoma, With Particular Reference to Preoperative Diagnosis, Value of Molecular Genetic Diagnosis, and Cell Origin. Surg Case Rep (2021) 7(1):208. doi: 10.1186/s40792-021-01295-4 34533614 PMC8448801

[29] Chen XY Rong DW Zhang L Ni CY Han GY Lu YW. Evaluation of Nodal Status in Intrahepatic Cholangiocarcinoma: A Population-Based Study. Ann Transl Med (2021) 9(17):1359. doi: 10.21037/atm-21-2785 34733911 PMC8506549

[30] Chen XY Lu YW Shi XL Chen XJ Rong DW Hang GY. Morbidity, Prognostic Factors, and Competing Risk Nomogram for Combined Hepatocellular-Cholangiocarcinoma. J Oncol (2021) 2021:3002480. doi: 10.1155/2021/3002480 34925507 PMC8683178

[31] Farooq A Merath K Paredes AZ Wu L Tsilimigras DI Hyer JM. Outcomes of Patients With Scirrhous Hepatocellular Carcinoma: Insights From the National Cancer Database. J Gastrointest Surg (2020) 24(5):1049–60. doi: 10.1007/s11605-019-04282-1 31243715

[32] Lee JH Choi MS Gwak GY Lee JH Koh KC Paik SW. Clinicopathologic Characteristics and Long-Term Prognosis of Scirrhous Hepatocellular Carcinoma. Dig Dis Sci (2012) 57(6):1698–707. doi: 10.1007/s10620-012-2075-x 22327241

[33] Kim SH Lim HK Lee WJ Choi D Park CK. Scirrhous Hepatocellular Carcinoma: Comparison With Usual Hepatocellular Carcinoma Based on CT-Pathologic Features and Long-Term Results After Curative Resection. Eur J Radiol (2009) 69(1):123–30. doi: 10.1016/j.ejrad.2007.09.008 17976942

[34] Solipuram V Baretti M Kim AY Chen LX Fahrner JA Gunay-Aygun M. Surgical Debulking for Refractory Hyperammonemic Encephalopathy in Fibrolamellar Hepatocellular Carcinoma. Hepatology (2021) 74(5):2899–901. doi: 10.1002/hep.31998 PMC9472764 34105830

[35] Surjan RCT Santos ESD Silveira SDP Makdissi FF Machado MAC. Fibrolamellar Hepatocellular Carcinoma-Related Hyperammonemic Encephalopathy: Up to Now and Next Steps. Clin Mol Hepatol (2020) 26(2):231–2. doi: 10.3350/cmh.2019.0084 PMC7160343 31679315

[36] Thakral N Simonetto DA. Hyperammonemic Encephalopathy: An Unusual Presentation of Fibrolamellar Hepatocellular Carcinoma. Clin Mol Hepatol (2020) 26(1):74–7. doi: 10.3350/cmh.2018.0042 PMC6940482 31422648

[37] Omata M Peters RL Tatter D. Sclerosing Hepatic Carcinoma: Relationship to Hypercalcemia. Liver (1981) 1(1):33–49. doi: 10.1111/j.1600-0676.1981.tb00020.x 6294435

[38] El Jabbour T Lagana SM Lee H. Update on Hepatocellular Carcinoma: Pathologists’ Review. World J Gastroenterol (2019) 25(14):1653–65. doi: 10.3748/wjg.v25.i14.1653 PMC6465943 31011252

[39] Calderaro J Couchy G Imbeaud S Amaddeo G Letouzé E Blanc JF. Histological Subtypes of Hepatocellular Carcinoma are Related to Gene Mutations and Molecular Tumour Classification. J Hepatol (2017) 67(4):727–38. doi: 10.1016/j.jhep.2017.05.014 28532995

[40] Limaiem F Bouraoui S Sboui M Bouslama S Lahmar A Mzabi S. Fibrolamellar Carcinoma Versus Scirrhous Hepatocellular Carcinoma: Diagnostic Usefulness of CD68. Acta Gastroenterol Belg (2015) 78(4):393–8. 26712049

[41] Honeyman JN Simon EP Robine N Chiaroni-Clarke R Darcy DG Lim II. Detection of a Recurrent DNAJB1-PRKACA Chimeric Transcript in Fibrolamellar Hepatocellular Carcinoma. Science (2014) 343(6174):1010–4. doi: 10.1126/science.1249484 PMC4286414 24578576

[42] Kirschner LS Carney JA Pack SD Taymans SE Giatzakis C Cho YS. Mutations of the Gene Encoding the Protein Kinase A Type I-Alpha Regulatory Subunit in Patients With the Carney Complex. Nat Genet (2000) 26(1):89–92. doi: 10.1038/79238 10973256

[43] Bannasch P Ribback S Su Q Mayer D. Clear Cell Hepatocellular Carcinoma: Origin, Metabolic Traits and Fate of Glycogenotic Clear and Ground Glass Cells. Hepatobiliary Pancreat Dis Int (2017) 16(6):570–94. doi: 10.1016/S1499-3872(17)60071-7 29291777

[44] Li Z Wu X Bi X Zhang Y Huang Z Lu H. Clinicopathological Features and Surgical Outcomes of Four Rare Subtypes of Primary Liver Carcinoma. Chin J Cancer Res (2018) 30(3):364–72. doi: 10.21147/j.issn.1000-9604.2018.03.08 PMC6037584 30046230

[45] Chen ZS Zhu SL Qi LN Li LQ. Long-Term Survival and Prognosis for Primary Clear Cell Carcinoma of the Liver After Hepatectomy. Onco Targets Ther (2016) 9:4129–35. doi: 10.2147/OTT.S104827 PMC4939989 27462167

[46] Xu W Ge P Liao W Ren J Yang H Xu H. Edmondson Grade Predicts Survival of Patients With Primary Clear Cell Carcinoma of Liver After Curative Resection: A Retrospective Study With Long-Term Follow-Up. Asia Pac J Clin Oncol (2017) 13(5):e312–20. doi: 10.1111/ajco.12494 27098441

[47] Liu Z Ma W Li H Li Q. Clinicopathological and Prognostic Features of Primary Clear Cell Carcinoma of the Liver. Hepatol Res (2008) 38(3):291–9. doi: 10.1111/j.1872-034X.2007.00264.x 17877725

[48] Morisue R Kojima M Suzuki T Nakatsura T Ojima H Watanabe R. Sarcomatoid Hepatocellular Carcinoma is Distinct From Ordinary Hepatocellular Carcinoma: Clinicopathologic, Transcriptomic and Immunologic Analyses. Int J Cancer (2021) 149(3):546–60. doi: 10.1002/ijc.33545 33662146

[49] Ji W Xing Y Ma J Zhao Z Xu H Zheng S. Primary Liver Sarcomatoid Carcinoma: A Case Series and Literature Review. J Hepatocell Carcinoma (2021) 8:1117–27. doi: 10.2147/JHC.S325182 PMC8434859 34522692

[50] Zhang C Feng S Tu Z Sun J Rui T Zhang X. Sarcomatoid Hepatocellular Carcinoma: From Clinical Features to Cancer Genome. Cancer Med (2021) 10(18):6227–38. doi: 10.1002/cam4.4162 PMC8446410 34331411

[51] Matak A Lahiri P Ford E Pabst D Kashofer K Stellas D. Stochastic Phenotype Switching Leads to Intratumor Heterogeneity in Human Liver Cancer. Hepatology (2018) 68(3):933–48. doi: 10.1002/hep.29679 PMC6175233 29171037

[52] De Sousa E Melo F Vermeulen L Fessler E Medema JP. Cancer Heterogeneity–A Multifaceted View. EMBO Rep (2013) 14(8):686–95. doi: 10.1038/embor.2013.92 PMC3736134 23846313

[53] Visvader JE Lindeman GJ. Cancer Stem Cells: Current Status and Evolving Complexities. Cell Stem Cell (2012) 10(6):717–28. doi: 10.1016/j.stem.2012.05.007 22704512

[54] Luo C Xin H Yin D Zhao T Hu Z Zhou Z. Characterization of Immune Infiltration in Sarcomatoid Hepatocellular Carcinoma. Aging (Albany NY) (2021) 13(11):15126–38. doi: 10.18632/aging.203076 PMC8221324 34081621

[55] Zhu SG Li HB Yuan ZN Liu W Yang Q Cheng Y. Achievement of Complete Response to Nivolumab in a Patient With Advanced Sarcomatoid Hepatocellular Carcinoma: A Case Report. World J Gastrointest Oncol (2020) 12(10):1209–15. doi: 10.4251/wjgo.v12.i10.1209 PMC7579730 33133387

[56] Yu Y Zhong Y Wang J Wu D. Sarcomatoid Hepatocellular Carcinoma (SHC): A Case Report. World J Surg Oncol (2017) 15(1):219. doi: 10.1186/s12957-017-1286-1 29233162 PMC5728015

[57] Huang Z Meng X Liu Q. Simultaneous Occurrence of Sarcomatoid Hepatocellular Carcinoma and Hepatocellular Carcinoma. J Cancer Res Ther (2015) 11(3):665. doi: 10.4103/0973-1482.140806 26458697

[58] Wang JP Yao ZG Sun YW Liu XH Sun FK Lin CH. Clinicopathological Characteristics and Surgical Outcomes of Sarcomatoid Hepatocellular Carcinoma. World J Gastroenterol (2020) 26(29):4327–42. doi: 10.3748/wjg.v26.i29.4327 PMC7422543 32848337

[59] Wu L Tsilimigras DI Farooq A Hyer JM Merath K Paredes AZ. Management and Outcomes Among Patients With Sarcomatoid Hepatocellular Carcinoma: A Population-Based Analysis. Cancer (2019) 125(21):3767–75. doi: 10.1002/cncr.32396 31299092

[60] Liao SH Su TH Jeng YM Liang PC Chen DS Chen CH. Clinical Manifestations and Outcomes of Patients With Sarcomatoid Hepatocellular Carcinoma. Hepatology (2019) 69(1):209–21. doi: 10.1002/hep.30162 30014620

