# Supplementary material for: Epidemiological and Clinical Characteristics of Five Rare Pathological Subtypes of Hepatocellular Carcinoma
Source: Front Oncol. 2022 Apr 8;12:864106. doi: 10.3389/fonc.2022.864106 (PMC9026181; doi:10.3389/fonc.2022.864106)
Supplement: Supplementary file 2 [file Image_2.pdf]

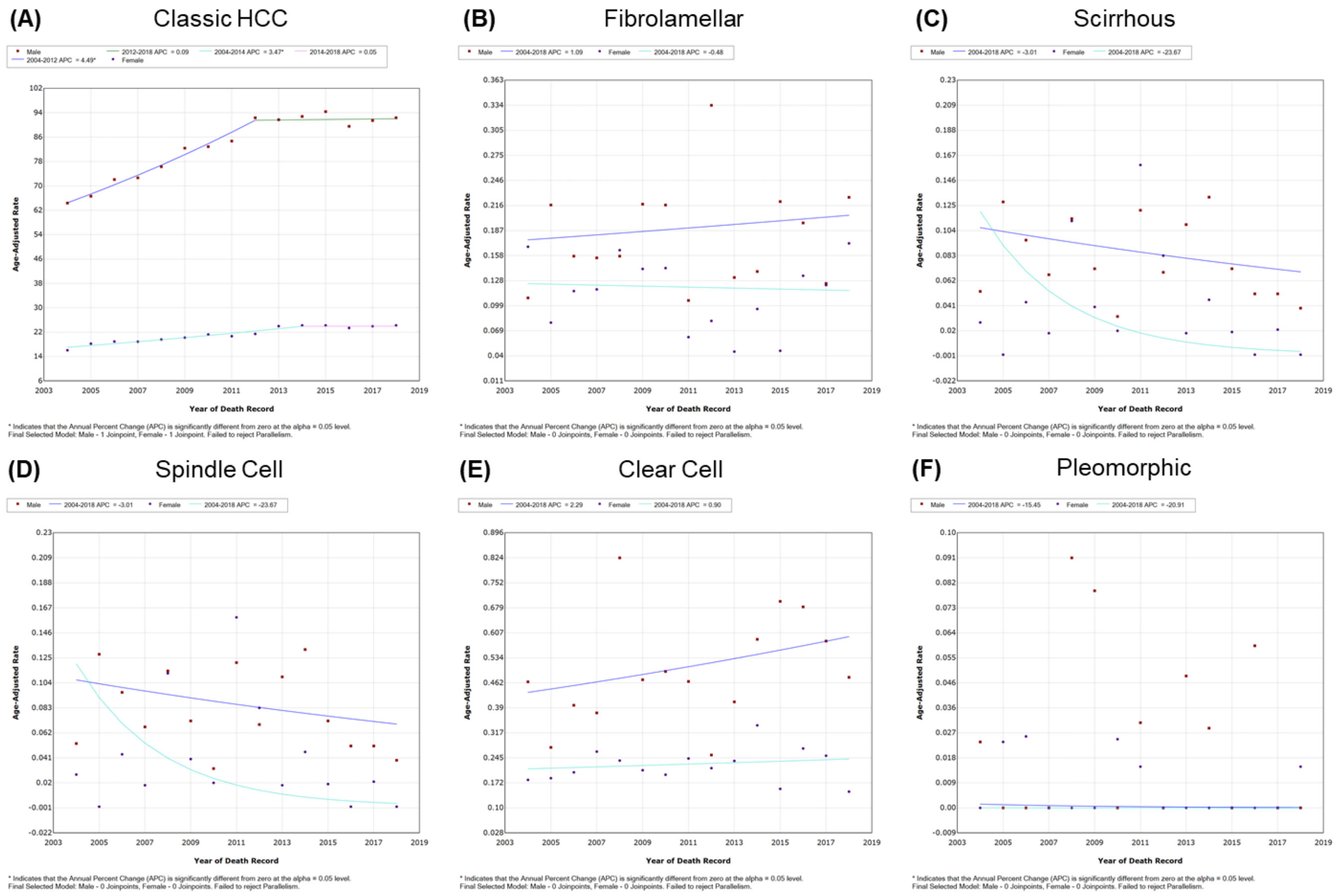

Figure S2. The variation trends for the gender-specific IBM of different pathological subtypes of HCC from 2004 to 2018. **(A)** Classic HCC; **(B)** Fibrolamellar carcinoma; **(C)** Scirrhous carcinoma; **(D)** Spindle cell carcinoma; **(E)** Clear cell carcinoma; **(F)** Pleomorphic carcinoma.

IBM, Incidence-based mortality; HCC, Hepatocellular carcinoma.
