# Supplementary material for: Epidemiological and Clinical Characteristics of Five Rare Pathological Subtypes of Hepatocellular Carcinoma
Source: Front Oncol. 2022 Apr 8;12:864106. doi: 10.3389/fonc.2022.864106 (PMC9026181; doi:10.3389/fonc.2022.864106)
Supplement: Supplementary file 3 [file Image_3.pdf]

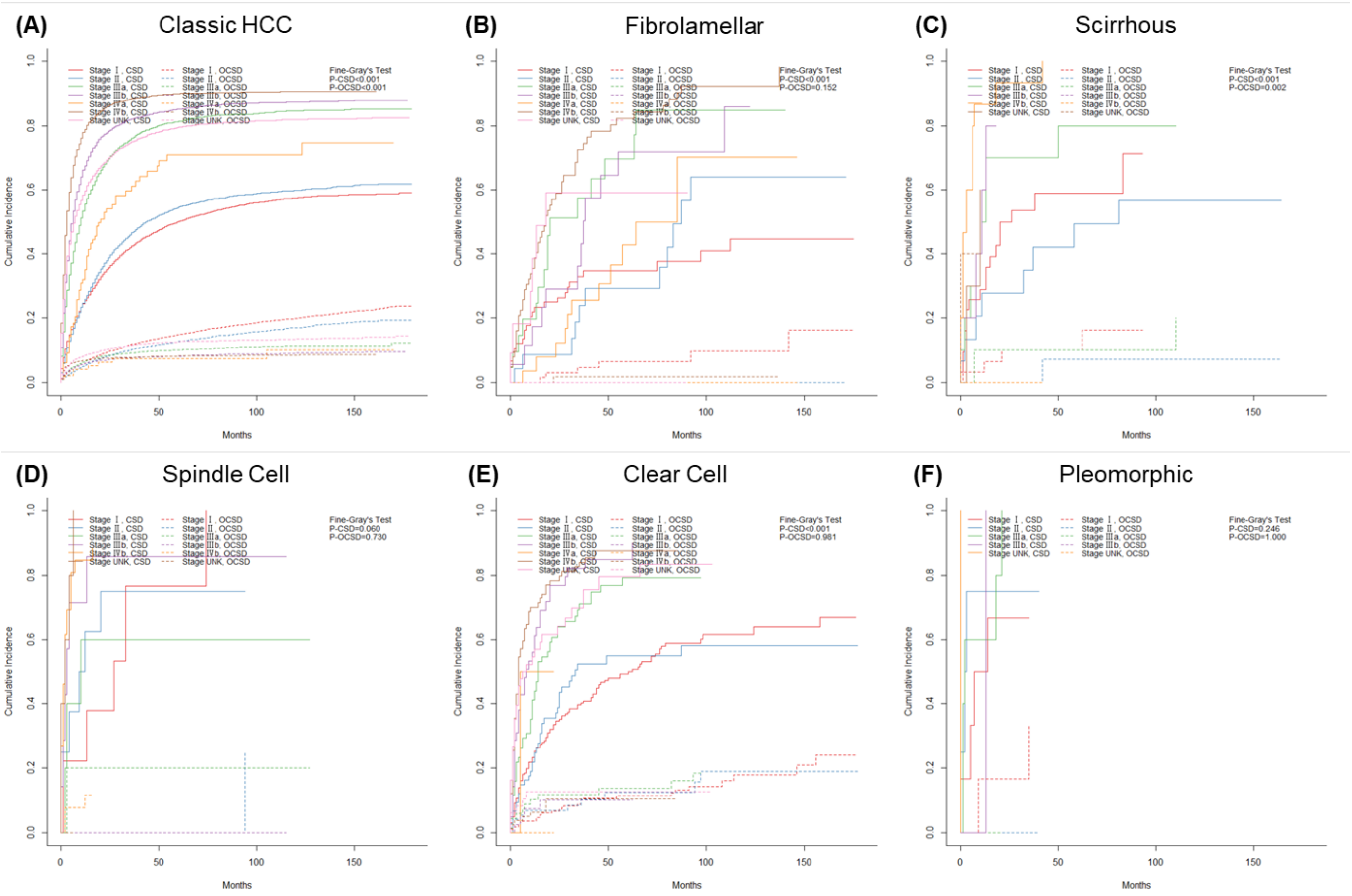

Figure S3. Cumulative incidence function curves of mortality in HCC patients stratified by different clinical stages. **(A)** Classic HCC; **(B)** Fibrolamellar carcinoma; **(C)** Scirrhous carcinoma; **(D)** Spindle cell carcinoma; **(E)** Clear cell carcinoma; **(F)** Pleomorphic carcinoma.

LND, Lymph node dissection; LNM, Lymph node metastasis; CSD, Cancer-specific death; OCSD, Other cause-specific death.
