# Supplementary material for: Epidemiological and Clinical Characteristics of Five Rare Pathological Subtypes of Hepatocellular Carcinoma
Source: Front Oncol. 2022 Apr 8;12:864106. doi: 10.3389/fonc.2022.864106 (PMC9026181; doi:10.3389/fonc.2022.864106)
Supplement: Supplementary file 5 [file Image_5.pdf]

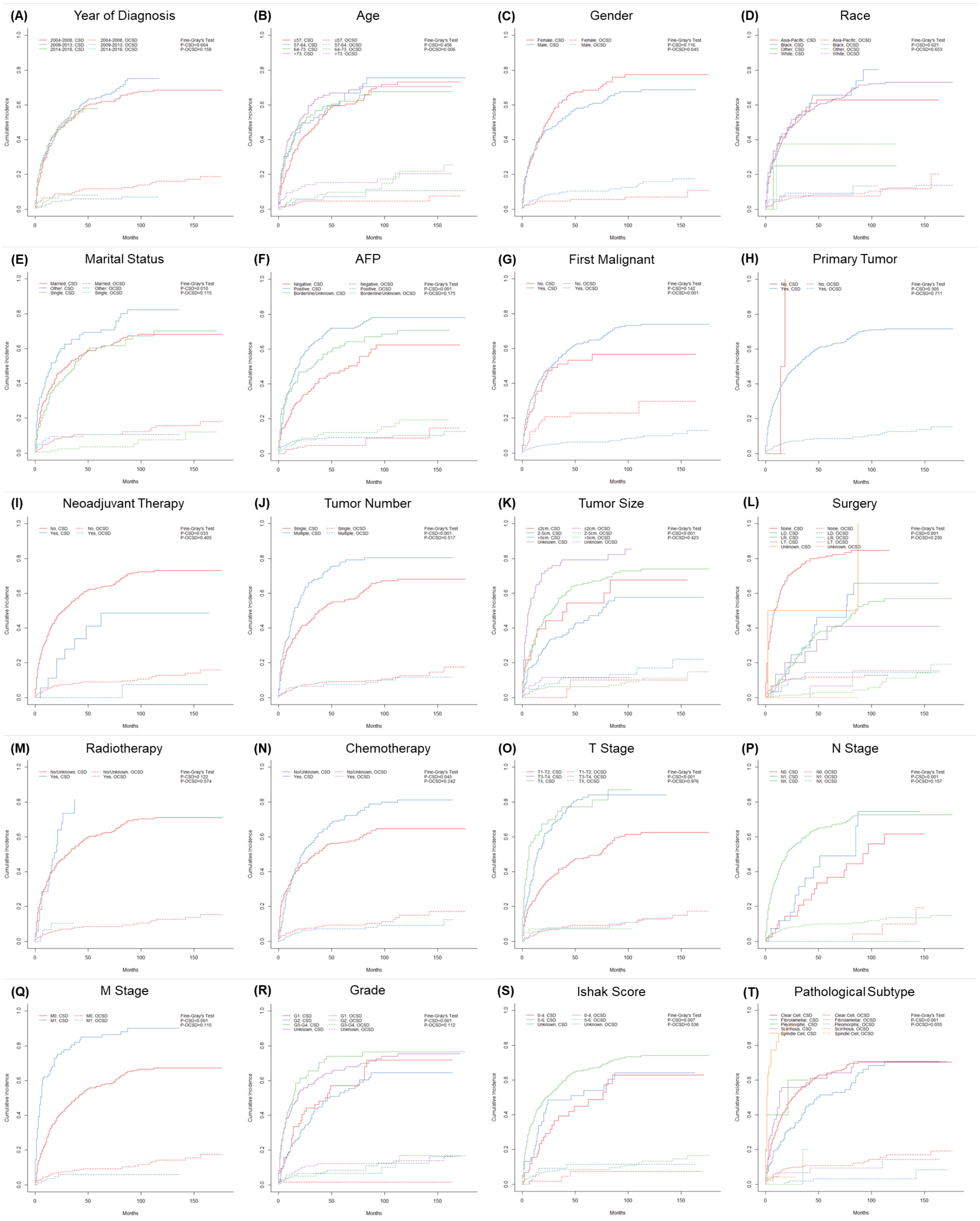

Figure S5. Cumulative incidence function curves of mortality in the training cohort stratified by different factors: **(A)** Year of diagnosis; **(B)** Age; **(C)** Gender; **(D)** Race; **(E)** Marital status; **(F)** AFP; **(G)** Cancer history; **(H)** Primary tumor; **(I)** Neoadjuvant therapy; **(J)** Tumor number; **(K)** Tumor size; **(L)** Surgery; **(M)** Radiotherapy; **(N)** Chemotherapy; **(O)** T stage; **(P)** N stage; **(Q)** M stage; **(R)** Grade; **(S)** Ishak score; **(T)** Pathological subtype.

AFP, Alpha-fetoprotein.
