# Supplementary material for: Epidemiological and Clinical Characteristics of Five Rare Pathological Subtypes of Hepatocellular Carcinoma
Source: Front Oncol. 2022 Apr 8;12:864106. doi: 10.3389/fonc.2022.864106 (PMC9026181; doi:10.3389/fonc.2022.864106)
Supplement: Supplementary file 6 [file Image_6.pdf]

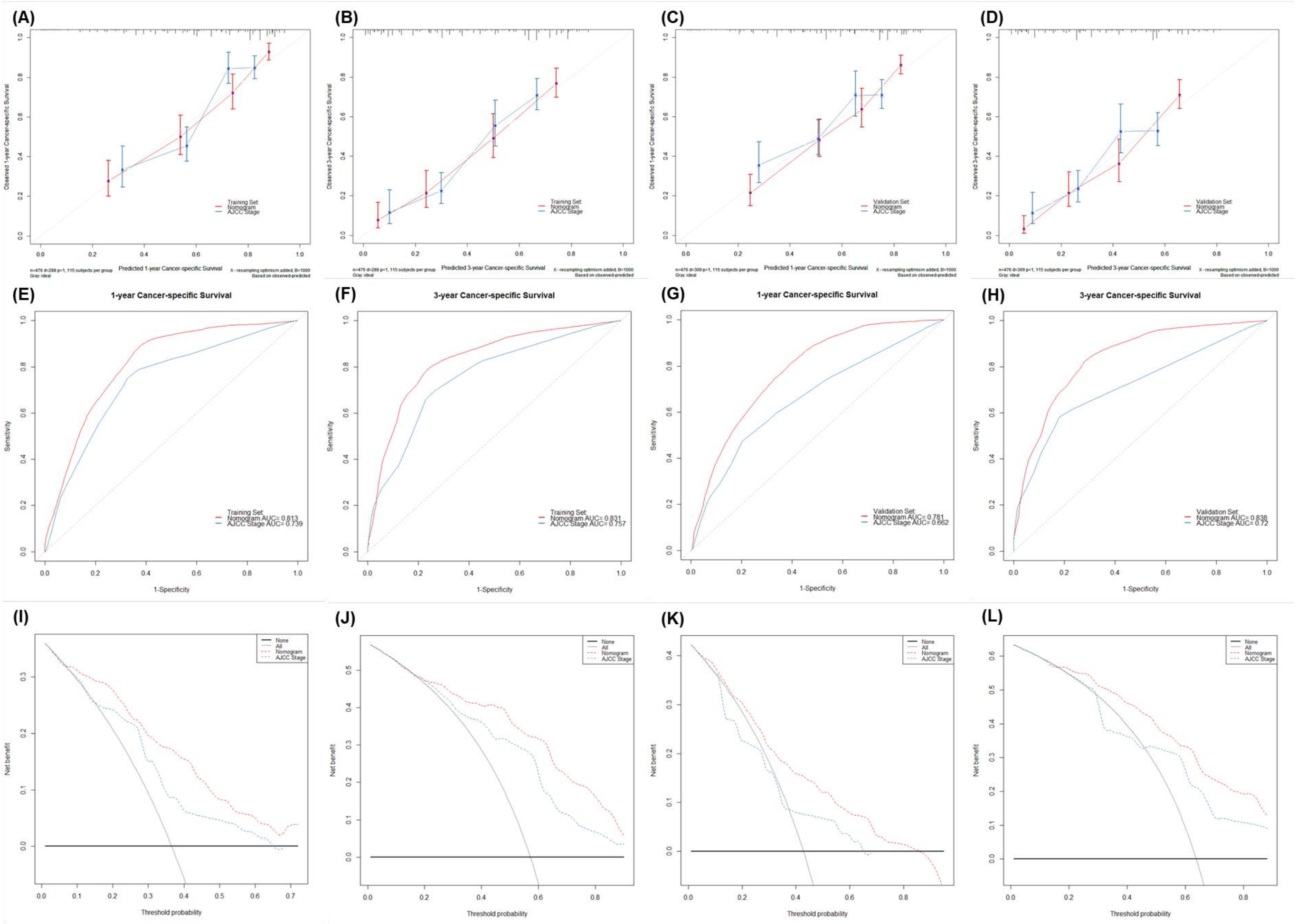

Figure S6. Validation of the novel predicting model developed from the training set. **(A)-(D)** Calibration curve analyses of the nomogram and the current AJCC staging system (8th edition) to evaluate prognoses effects at 1- and 3-year points in the training and validation sets. **(E)-(H)** Receiver operating characteristic curve analyses of the nomogram and the current AJCC staging system (8th edition) to evaluate prognoses effects at 1- and 3-year points in the training and validation sets. **(I)-(L)** Decision curve analyses of the nomogram and the current AJCC staging system (8th edition) to evaluate prognoses effects at 1- and 3-year points in the training and validation sets.

LT, Liver transplantation; LR, Liver resection; LD, Local destruction; UNK, Unknown; AFP, Alpha-fetoprotein.
