# Supplementary material for: Epidemiological and Clinical Characteristics of Five Rare Pathological Subtypes of Hepatocellular Carcinoma
Source: Front Oncol. 2022 Apr 8;12:864106. doi: 10.3389/fonc.2022.864106 (PMC9026181; doi:10.3389/fonc.2022.864106)
Supplement: Supplementary file 7 [file Table_1.docx]

**Supplementary Tables**

| Contents | | |
| --- | --- | --- |
| Table | Legends | Page |
| S1 | The P-values for factors compared to those of classic HCC | 2 |
| S2 | Comparison between LND and Non-LND before and after PSM in patients with classic HCC | 3 |
| S3 | Comparison between LND and Non-LND before and after PSM in patients with fibrolamellar carcinoma | 5 |
| S4 | Comparison between LND and Non-LND before and after PSM in patients with clear cell carcinoma | 7 |
| S5 | Comparison between LNM and Non-LNM before and after PSM in patients with classic HCC | 9 |
| S6 | Comparison between LNM and Non-LNM before and after PSM in patients with fibrolamellar carcinoma | 11 |
| S7 | Case series of LT recipients with rare pathological subtypes of HCC | 13 |
| S8 | Baseline characteristics of patients with rare pathological subtypes of HCC in the training and validation sets | 14 |
| S9 | Competing risk survival analyses of patients with rare pathological subtypes of HCC | 16 |
| S10 | Cumulative incidence of CSD and OCSD of patients with rare pathological subtypes of HCC | 18 |

**Table S1**. The P-values for factors compared to those of classic HCC

| Factors | Fibrolamellar  (n=241) | Scirrhous  (n=82) | Spindle Cell  (n=61) | Clear Cell  (n=551) | Pleomorphic  (n=17) |
| --- | --- | --- | --- | --- | --- |
| Year of Diagnosis | 0.169 | 0.007 | 0.099 | 0.252 | 0.215 |
| Age | <0.001 | 0.290 | 0.995 | <0.001 | 0.295 |
| Gender | <0.001 | 0.005 | 0.642 | <0.001 | 0.727 |
| Race | 0.001 | 0.120 | 0.780 | 0.006 | 0.651 |
| Marital Status | <0.001 | 0.281 | 0.855 | 0.001 | 0.272 |
| AFP | <0.001 | 0.609 | 0.955 | 0.087 | 0.112 |
| First Malignant | <0.001 | 1.000 | 0.502 | 0.006 | 0.155 |
| Primary Tumor | 0.819 | 1.000 | 1.000 | 1.000 | 1.000 |
| Neoadjuvant Therapy | 0.111 | 0.885 | 1.000 | 0.104 | 1.000 |
| Tumor Number | 0.117 | 0.801 | 0.123 | 0.439 | 0.243 |
| Tumor Size | <0.001 | 0.484 | 0.080 | <0.001 | 0.033 |
| Surgery | <0.001 | 0.640 | 0.443 | <0.001 | 0.037 |
| Radiotherapy | 0.791 | 0.853 | 0.739 | 0.968 | 1.000 |
| Chemotherapy | <0.001 | 0.250 | 0.127 | 0.220 | 0.083 |
| T Stage | <0.001 | 0.634 | 0.051 | 0.005 | 0.365 |
| N Stage | <0.001 | 0.013 | 0.321 | 0.542 | 0.937 |
| M stage | <0.001 | 0.877 | <0.001 | 0.242 | 0.122 |
| Grade | 0.025 | 0.373 | <0.001 | 0.001 | <0.001 |
| Ishak Score | <0.001 | 0.881 | 0.172 | <0.001 | 0.676 |
| CSD | <0.001 | 0.713 | <0.001 | 0.171 | 0.015 |
| OCSD | <0.001 | 0.601 | 0.328 | 0.874 | 0.908 |

HCC, Hepatocellular carcinoma; AFP, Alpha fetoprotein; CSD, Cancer-specific death; OCSD, Other cause-specific death.

**Table S2**. Comparison between LND and Non-LND before and after PSM in patients with classic HCC

| Factors | Before PSM | | | After PSM | | |
| --- | --- | --- | --- | --- | --- | --- |
|  | LND  (n=2254) | Non-LND  (n=47012) | P | LND  (n=2249) | Non-LND  (n=2249) | P |
| Year of Diagnosis |  |  | 0.159 |  |  | 0.163 |
| 2004-2008 | 691(30.7) | 14248(30.3) |  | 689(30.6) | 685(30.5) |  |
| 2009-2013 | 735(32.6) | 16208(34.5) |  | 733(32.6) | 788(35.0) |  |
| 2014-2018 | 828(36.7) | 16556(35.2) |  | 827(36.8) | 776(34.5) |  |
| Age |  |  | <0.001 |  |  | 0.396 |
| ≤57 | 865(38.4) | 12258(26.1) |  | 862(38.3) | 865(38.5) |  |
| 57-64 | 702(31.1) | 11543(24.6) |  | 700(31.1) | 700(31.1) |  |
| 64-73 | 503(22.3) | 11659(24.8) |  | 503(22.4) | 472(21.0) |  |
| >73 | 184(8.2) | 11552(24.6) |  | 184(8.2) | 212(9.4) |  |
| Gender |  |  | 0.050 |  |  | 0.465 |
| Female | 555(24.6) | 10730(22.8) |  | 555(24.7) | 533(23.7) |  |
| Male | 1699(75.4) | 36282(77.2) |  | 1694(75.3) | 1716(76.3) |  |
| Race |  |  | 0.485 |  |  | 0.405 |
| White | 1571(69.7) | 32240(68.6) |  | 1567(69.7) | 1579(70.2) |  |
| Asia-Pacific | 353(15.7) | 7345(15.6) |  | 353(15.7) | 375(16.7) |  |
| Black | 298(13.2) | 6644(14.1) |  | 297(13.2) | 262(11.6) |  |
| Other | 32(1.4) | 783(1.7) |  | 32(1.4) | 33(1.5) |  |
| Marital Status |  |  | <0.001 |  |  | 0.220 |
| Married | 1412(62.6) | 24610(52.3) |  | 1408(62.6) | 1464(65.1) |  |
| Single | 383(17.0) | 9214(19.6) |  | 383(17.0) | 359(16.0) |  |
| Other | 459(20.4) | 13188(28.1) |  | 458(20.4) | 426(18.9) |  |
| AFP |  |  | <0.001 |  |  | 0.798 |
| Negative | 620(27.5) | 9626(20.5) |  | 620(27.6) | 622(27.7) |  |
| Positive | 1138(50.5) | 24100(51.3) |  | 1134(50.4) | 1115(49.6) |  |
| Borderline/Unknown | 496(22.0) | 13286(28.3) |  | 495(22.0) | 512(22.8) |  |
| First Malignant |  |  | <0.001 |  |  | 0.092 |
| Yes | 1994(88.5) | 39683(84.4) |  | 1990(88.5) | 2026(90.1) |  |
| No | 260(11.5) | 7329(15.6) |  | 259(11.5) | 223(9.9) |  |
| Primary Tumor |  |  | 0.142 |  |  | 1.000 |
| Yes | 2237(99.2) | 46775(99.5) |  | 2232(99.2) | 2233(99.3) |  |
| No | 17(0.8) | 237(0.5) |  | 17(0.8) | 16(0.7) |  |
| Neoadjuvant Therapy |  |  | <0.001 |  |  | 0.522 |
| Yes | 453(20.1) | 926(2.0) |  | 448(19.9) | 430(19.1) |  |
| No | 1801(79.9) | 46086(98.0) |  | 1801(80.1) | 1819(80.9) |  |
| Tumor Number |  |  | 0.057 |  |  | 0.468 |
| Single | 1596(70.8) | 32384(68.9) |  | 1593(70.8) | 1616(71.9) |  |
| Multiple | 658(29.2) | 14628(31.1) |  | 656(29.2) | 633(28.1) |  |
| Tumor Size |  |  | <0.001 |  |  | 0.813 |
| ≤2cm | 476(21.1) | 4103(8.7) |  | 476(21.2) | 466(20.7) |  |
| 2-5cm | 990(43.9) | 14960(31.8) |  | 989(44.0) | 989(44.0) |  |
| ＞5cm | 747(33.1) | 18261(38.8) |  | 743(33.0) | 760(33.8) |  |
| Unknown | 41(1.8) | 9688(20.6) |  | 41(1.8) | 34(1.5) |  |
| Surgery |  |  | <0.001 |  |  | 0.626 |
| None | 0(0) | 32650(69.5) |  | 0(0) | 0(0) |  |
| LD | 61(2.7) | 4877(10.4) |  | 61(2.7) | 64(2.8) |  |
| LR | 1032(45.8) | 6282(13.4) |  | 1032(45.9) | 988(43.9) |  |
| LT | 1151(51.1) | 2709(5.8) |  | 1146(51.0) | 1187(52.8) |  |
| Method Unknown | 10(0.4) | 494(1.1) |  | 10(0.4) | 10(0.4) |  |
| Radiotherapy |  |  | <0.001 |  |  | 1.000 |
| Yes | 87(3.9) | 4460(9.5) |  | 86(3.8) | 86(3.8) |  |
| No/Unknown | 2167(96.1) | 42552(90.5) |  | 2163(96.2) | 2163(96.2) |  |
| Chemotherapy |  |  | 0.006 |  |  | 1.000 |
| Yes | 721(32.0) | 16383(34.8) |  | 716(31.8) | 717(31.9) |  |
| No/Unknown | 1533(68.0) | 30629(65.2) |  | 1533(68.2) | 1532(68.1) |  |
| T Stage |  |  | <0.001 |  |  | 0.981 |
| T1a | 314(13.9) | 2754(5.9) |  | 314(14.0) | 314(14.0) |  |
| T1b | 784(34.8) | 13885(29.5) |  | 784(34.9) | 797(35.4) |  |
| T1NOS | 20(0.9) | 2105(4.5) |  | 20(0.9) | 16(0.7) |  |
| T2 | 716(31.8) | 8100(17.2) |  | 715(31.8) | 718(31.9) |  |
| T3 | 201(8.9) | 6831(14.5) |  | 200(8.9) | 186(8.3) |  |
| T4 | 198(8.8) | 5677(12.1) |  | 195(8.7) | 198(8.8) |  |
| TX | 21(0.9) | 7660(16.3) |  | 21(0.9) | 20(0.9) |  |
| M Stage |  |  | <0.001 |  |  | 1.000 |
| M0 | 2207(97.9) | 38664(82.2) |  | 2202(97.9) | 2202(97.9) |  |
| M1 | 47(2.1) | 8348(17.8) |  | 47(2.1) | 47(2.1) |  |
| Grade^†^ |  |  | <0.001 |  |  | 0.806 |
| G1 | 467(20.7) | 8727(18.6) |  | 466(20.7) | 486(21.6) |  |
| G2 | 967(42.9) | 12209(26.0) |  | 965(42.9) | 945(42.0) |  |
| G3-G4 | 416(18.5) | 6363(13.5) |  | 414(18.4) | 402(17.9) |  |
| Unknown | 404(17.9) | 19713(41.9) |  | 404(18.0) | 416(18.5) |  |
| Ishak Score |  |  | <0.001 |  |  | 0.414 |
| 0-4 | 299(13.3) | 2836(6.0) |  | 297(13.2) | 275(12.2) |  |
| 5-6 | 670(29.7) | 7351(15.6) |  | 669(29.7) | 650(28.9) |  |
| Unknown | 1285(57.0) | 36825(78.3) |  | 1283(57.0) | 1324(58.9) |  |

HCC, Hepatocellular carcinoma; LND, Lymph node dissection; PSM, Propensity score matching; AFP, Alpha-fetoprotein; LD, Local destruction; LR, Liver resection; LT, Liver transplantation.

^†^G1=Well differentiated; G2=Moderately differentiated; G3-4=Poorly differentiated/Undifferentiated.

**Table S3**. Comparison between LND and Non-LND before and after PSM in patients with fibrolamellar carcinoma

| Factors | Before PSM | | | After PSM | | |
| --- | --- | --- | --- | --- | --- | --- |
|  | LND  (n=80) | Non-LND  (n=161) | P | LND  (n=35) | Non-LND  (n=35) | P |
| Year of Diagnosis |  |  | 0.798 |  |  | 0.694 |
| 2004-2008 | 30(37.5) | 56(34.8) |  | 15(42.9) | 14(40.0) |  |
| 2009-2013 | 22(27.5) | 51(31.7) |  | 7(20.0) | 10(28.6) |  |
| 2014-2018 | 28(35.0) | 54(33.5) |  | 13(37.1) | 11(31.4) |  |
| Age |  |  | <0.001 |  |  | - |
| ≤57 | 79(98.8) | 117(72.7) |  | 35(100.0) | 35(100.0) |  |
| 57-64 | 0(0) | 15(9.3) |  | 0(0) | 0(0) |  |
| 64-73 | 1(1.2) | 15(9.3) |  | 0(0) | 0(0) |  |
| >73 | 0(0) | 14(8.7) |  | 0(0) | 0(0) |  |
| Gender |  |  | 0.452 |  |  | 0.332 |
| Female | 29(36.2) | 68(42.2) |  | 12(34.3) | 17(48.6) |  |
| Male | 51(63.7) | 93(57.8) |  | 23(65.7) | 18(51.4) |  |
| Race |  |  | 0.709 |  |  | 0.826 |
| White | 66(82.5) | 128(79.5) |  | 27(77.1) | 29(82.9) |  |
| Asia-Pacific | 7(8.8) | 13(8.1) |  | 3(8.6) | 2(5.7) |  |
| Black | 7(8.8) | 18(11.2) |  | 5(14.3) | 4(11.4) |  |
| Other | 0(0) | 2(1.2) |  | 0(0) | 0(0) |  |
| Marital Status |  |  | 0.215 |  |  | 0.412 |
| Married | 18(22.5) | 51(31.7) |  | 11(31.4) | 7(20.0) |  |
| Single | 56(70.0) | 94(58.4) |  | 24(68.6) | 28(80.0) |  |
| Other | 6(7.5) | 16(9.9) |  | 0(0) | 0(0) |  |
| AFP |  |  | 0.002 |  |  | 0.846 |
| Negative | 47(58.8) | 60(37.3) |  | 19(54.3) | 17(48.6) |  |
| Positive | 14(17.5) | 60(37.3) |  | 8(22.9) | 8(22.9) |  |
| Borderline/Unknown | 19(23.8) | 41(25.5) |  | 8(22.9) | 10(28.6) |  |
| First Malignant |  |  | 0.027 |  |  | - |
| Yes | 79(98.8) | 145(90.1) |  | 35(100.0) | 35(100.0) |  |
| No | 1(1.2) | 16(9.9) |  | 0(0) | 0(0) |  |
| Primary Tumor |  |  | 0.805 |  |  | - |
| Yes | 80(100.0) | 159(98.8) |  | 35(100.0) | 35(100.0) |  |
| No | 0(0) | 2(1.2) |  | 0(0) | 0(0) |  |
| Neoadjuvant Therapy |  |  | 0.027 |  |  | 1.000 |
| Yes | 8(10.0) | 4(2.5) |  | 2(5.7) | 3(8.6) |  |
| No | 72(90.0) | 157(97.5) |  | 33(94.3) | 32(91.4) |  |
| Tumor Number |  |  | 0.092 |  |  | 1.000 |
| Single | 15(18.8) | 48(29.8) |  | 29(82.9) | 28(80.0) |  |
| Multiple | 65(81.2) | 113(70.2) |  | 6(17.1) | 7(20.0) |  |
| Tumor Size |  |  | 0.003 |  |  | 0.588 |
| ≤2cm | 2(2.5) | 8(5.0) |  | 0(0) | 0(0) |  |
| 2-5cm | 5(6.2) | 25(15.5) |  | 1(2.9) | 3(8.6) |  |
| ＞5cm | 69(86.2) | 102(63.4) |  | 33(94.3) | 31(88.6) |  |
| Unknown | 4(5.0) | 26(16.1) |  | 1(2.9) | 1(2.9) |  |
| Surgery |  |  | <0.001 |  |  | 1.000 |
| None | 0(0) | 99(61.5) |  | 0(0) | 0(0) |  |
| LD | 0(0) | 5(3.1) |  | 0(0) | 0(0) |  |
| LR | 70(87.5) | 52(32.3) |  | 31(88.6) | 31(88.6) |  |
| LT | 9(11.2) | 4(2.5) |  | 4(11.4) | 4(11.4) |  |
| Method Unknown | 1(1.2) | 1(0.6) |  | 0(0) | 0(0) |  |
| Radiotherapy |  |  | 0.144 |  |  | 1.000 |
| Yes | 4(5.0) | 19(11.8) |  | 2(5.7) | 2(5.7) |  |
| No/Unknown | 76(95.0) | 142(88.2) |  | 33(94.3) | 33(94.3) |  |
| Chemotherapy |  |  | 0.022 |  |  | 1.000 |
| Yes | 30(37.5) | 87(54.0) |  | 12(34.3) | 13(37.1) |  |
| No/Unknown | 50(62.5) | 74(46.0) |  | 23(65.7) | 22(62.9) |  |
| T Stage |  |  | 0.032 |  |  | 0.882 |
| T1a | 0(0) | 7(4.3) |  | 0(0) | 0(0) |  |
| T1b | 34(42.5) | 51(31.7) |  | 17(48.6) | 15(42.9) |  |
| T1NOS | 2(2.5) | 6(3.7) |  | 0(0) | 0(0) |  |
| T2 | 13(16.2) | 22(13.7) |  | 7(20.0) | 9(25.7) |  |
| T3 | 12(15.0) | 30(18.6) |  | 4(11.4) | 6(17.1) |  |
| T4 | 18(22.5) | 27(16.8) |  | 6(17.1) | 4(11.4) |  |
| TX | 1(1.2) | 18(11.2) |  | 1(2.9) | 1(2.9) |  |
| M Stage |  |  | 0.092 |  |  | 1.000 |
| M0 | 65(81.2) | 113(70.2) |  | 31(88.6) | 31(88.6) |  |
| M1 | 15(18.8) | 48(29.8) |  | 4(11.4) | 4(11.4) |  |
| Grade^†^ |  |  | 0.004 |  |  | 0.795 |
| G1 | 8(10.0) | 14(8.7) |  | 7(20.0) | 5(14.3) |  |
| G2 | 31(38.8) | 30(18.6) |  | 9(25.7) | 7(20.0) |  |
| G3-G4 | 8(10.0) | 16(9.9) |  | 3(8.6) | 3(8.6) |  |
| Unknown | 33(41.2) | 101(62.7) |  | 16(45.7) | 20(57.1) |  |
| Ishak Score |  |  | 0.041 |  |  | 0.539 |
| 0-4 | 18(22.5) | 19(11.8) |  | 5(14.3) | 8(22.9) |  |
| 5-6 | 0(0) | 4(2.5) |  | 0(0) | 0(0) |  |
| Unknown | 62(77.5) | 138(85.7) |  | 30(85.7) | 27(77.1) |  |

LND, Lymph node dissection; PSM, Propensity score matching; AFP, Alpha-fetoprotein; LD, Local destruction; LR, Liver resection; LT, Liver transplantation.

^†^G1=Well differentiated; G2=Moderately differentiated; G3-4=Poorly differentiated/Undifferentiated.

**Table S4**. Comparison between LND and Non-LND before and after PSM in patients with clear cell carcinoma

| Factors | Before PSM | | | After PSM | | |
| --- | --- | --- | --- | --- | --- | --- |
|  | LND  (n=30) | Non-LND  (n=521) | P | LND  (n=22) | Non-LND  (n=22) | P |
| Year of Diagnosis |  |  | 0.980 |  |  | 0.937 |
| 2004-2008 | 8(26.7) | 144(27.6) |  | 6(27.3) | 6(27.3) |  |
| 2009-2013 | 10(33.3) | 178(34.2) |  | 6(27.3) | 7(31.8) |  |
| 2014-2018 | 12(40.0) | 199(38.2) |  | 10(45.5) | 9(40.9) |  |
| Age |  |  | 0.015 |  |  | 0.721 |
| ≤57 | 9(30.0) | 101(19.4) |  | 7(31.8) | 5(22.7) |  |
| 57-64 | 11(36.7) | 101(19.4) |  | 9(40.9) | 9(40.9) |  |
| 64-73 | 7(23.3) | 156(29.9) |  | 5(22.7) | 5(22.7) |  |
| >73 | 3(10.0) | 163(31.3) |  | 1(4.5) | 3(13.6) |  |
| Gender |  |  | 0.180 |  |  | 0.763 |
| Female | 15(50.0) | 188(36.1) |  | 12(54.5) | 10(45.5) |  |
| Male | 15(50.0) | 333(63.9) |  | 10(45.5) | 12(54.5) |  |
| Race |  |  | 0.662 |  |  | 0.688 |
| White | 21(70.0) | 353(67.8) |  | 15(68.2) | 16(72.7) |  |
| Asia-Pacific | 4(13.3) | 107(20.5) |  | 4(18.2) | 5(22.7) |  |
| Black | 4(13.3) | 53(10.2) |  | 2(9.1) | 1(4.5) |  |
| Other | 1(3.3) | 8(1.5) |  | 1(4.5) | 0(0) |  |
| Marital Status |  |  | 0.489 |  |  | 0.580 |
| Married | 21(70.0) | 311(59.7) |  | 16(72.7) | 13(59.1) |  |
| Single | 4(13.3) | 77(14.8) |  | 2(9.1) | 4(18.2) |  |
| Other | 5(16.7) | 133(25.5) |  | 4(18.2) | 5(22.7) |  |
| AFP |  |  | 0.047 |  |  | 0.951 |
| Negative | 12(40.0) | 123(23.6) |  | 9(40.9) | 10(45.5) |  |
| Positive | 15(50.0) | 260(49.9) |  | 11(50.0) | 10(45.5) |  |
| Borderline/Unknown | 3(10.0) | 138(26.5) |  | 2(9.1) | 2(9.1) |  |
| First Malignant |  |  | 0.251 |  |  | 1.000 |
| Yes | 27(90.0) | 415(79.7) |  | 19(86.4) | 19(86.4) |  |
| No | 3(10.0) | 106(20.3) |  | 3(13.6) | 3(13.6) |  |
| Primary Tumor |  |  | 1.000 |  |  | - |
| Yes | 30(100.0) | 518(99.4) |  | 22(100.0) | 22(100.0) |  |
| No | 0(0) | 3(0.6) |  | 0(0) | 0(0) |  |
| Neoadjuvant Therapy |  |  | 0.095 |  |  | 1.000 |
| Yes | 2(6.7) | 6(1.2) |  | 1(4.5) | 2(9.1) |  |
| No | 28(93.3) | 515(98.8) |  | 21(95.5) | 20(90.9) |  |
| Tumor Number |  |  | 0.171 |  |  | 1.000 |
| Single | 25(83.3) | 364(69.9) |  | 18(81.8) | 18(81.8) |  |
| Multiple | 5(16.7) | 157(30.1) |  | 4(18.2) | 4(18.2) |  |
| Tumor Size |  |  | 0.035 |  |  | 0.793 |
| ≤2cm | 2(6.7) | 31(6.0) |  | 2(9.1) | 2(9.1) |  |
| 2-5cm | 5(16.7) | 147(28.2) |  | 4(18.2) | 4(18.2) |  |
| ＞5cm | 22(73.3) | 250(48.0) |  | 16(72.7) | 15(68.2) |  |
| Unknown | 1(3.3) | 93(17.9) |  | 0(0) | 1(4.5) |  |
| Surgery |  |  | <0.001 |  |  | 1.000 |
| None | 0(0) | 330(63.3) |  | 0(0) | 0(0) |  |
| LD | 0(0) | 45(8.6) |  | 0(0) | 0(0) |  |
| LR | 25(83.3) | 134(25.7) |  | 19(86.4) | 18(81.8) |  |
| LT | 5(16.7) | 9(1.7) |  | 3(13.6) | 4(18.2) |  |
| Method Unknown | 0(0) | 3(0.6) |  | 0(0) | 0(0) |  |
| Radiotherapy |  |  | 0.408 |  |  | - |
| Yes | 1(3.3) | 50(9.6) |  | 0(0) | 0(0) |  |
| No/Unknown | 29(96.7) | 471(90.4) |  | 22(100.0) | 22(100.0) |  |
| Chemotherapy |  |  | 0.956 |  |  | 1.000 |
| Yes | 9(30.0) | 168(32.2) |  | 5(22.7) | 6(27.3) |  |
| No/Unknown | 21(70.0) | 353(67.8) |  | 17(77.3) | 16(72.7) |  |
| T Stage |  |  | 0.147 |  |  | 0.953 |
| T1a | 1(3.3) | 24(4.6) |  | 1(4.5) | 1(4.5) |  |
| T1b | 17(56.7) | 184(35.3) |  | 13(59.1) | 13(59.1) |  |
| T1NOS | 1(3.3) | 25(4.8) |  | 0(0) | 1(4.5) |  |
| T2 | 6(20.0) | 76(14.6) |  | 5(22.7) | 4(18.2) |  |
| T3 | 3(10.0) | 83(15.9) |  | 2(9.1) | 2(9.1) |  |
| T4 | 2(6.7) | 62(11.9) |  | 1(4.5) | 1(4.5) |  |
| TX | 0(0) | 67(12.9) |  | 0(0) | 0(0) |  |
| M Stage |  |  | 0.593 |  |  | 1.000 |
| M0 | 27(90.0) | 441(84.6) |  | 21(95.5) | 21(95.5) |  |
| M1 | 3(10.0) | 80(15.4) |  | 1(4.5) | 1(4.5) |  |
| Grade^†^ |  |  | 0.060 |  |  | 1.000 |
| G1 | 5(16.7) | 73(14.0) |  | 5(22.7) | 5(22.7) |  |
| G2 | 16(53.3) | 165(31.7) |  | 12(54.5) | 12(54.5) |  |
| G3-G4 | 2(6.7) | 73(14.0) |  | 2(9.1) | 2(9.1) |  |
| Unknown | 7(23.3) | 210(40.3) |  | 3(13.6) | 3(13.6) |  |
| Ishak Score |  |  | 0.012 |  |  | 0.748 |
| 0-4 | 9(30.0) | 60(11.5) |  | 6(27.3) | 4(18.2) |  |
| 5-6 | 3(10.0) | 65(12.5) |  | 3(13.6) | 4(18.2) |  |
| Unknown | 18(60.0) | 396(76.0) |  | 13(59.1) | 14(63.6) |  |

LND, Lymph node dissection; PSM, Propensity score matching; AFP, Alpha-fetoprotein; LD, Local destruction; LR, Liver resection; LT, Liver transplantation.

^†^G1=Well differentiated; G2=Moderately differentiated; G3-4=Poorly differentiated/Undifferentiated.

**Table S5**. Comparison between LNM and Non-LNM before and after PSM in patients with classic HCC

| Factors | Before PSM | | | After PSM | | |
| --- | --- | --- | --- | --- | --- | --- |
|  | LNM  (n=107) | Non-LNM  (n=2147) | P | LNM  (n=100) | Non-LNM  (n=100) | P |
| Year of Diagnosis |  |  | 0.163 |  |  | 0.866 |
| 2004-2008 | 41(38.3) | 650(30.3) |  | 38(38.0) | 35(35.0) |  |
| 2009-2013 | 28(26.2) | 707(32.9) |  | 26(26.0) | 29(29.0) |  |
| 2014-2018 | 38(35.5) | 790(36.8) |  | 36(36.0) | 36(36.0) |  |
| Age |  |  | 0.026 |  |  | 0.689 |
| ≤57 | 35(32.7) | 830(38.7) |  | 31(31.0) | 39(39.0) |  |
| 57-64 | 32(29.9) | 670(31.2) |  | 31(31.0) | 28(28.0) |  |
| 64-73 | 23(21.5) | 480(22.4) |  | 22(22.0) | 20(20.0) |  |
| >73 | 17(15.9) | 167(7.8) |  | 16(16.0) | 13(13.0) |  |
| Gender |  |  | 0.100 |  |  | 0.876 |
| Female | 34(31.8) | 521(24.3) |  | 30(30.0) | 28(28.0) |  |
| Male | 73(68.2) | 1626(75.7) |  | 70(70.0) | 72(72.0) |  |
| Race |  |  | 0.777 |  |  | 0.721 |
| White | 77(72.0) | 1494(69.6) |  | 70(70.0) | 72(72.0) |  |
| Asia-Pacific | 18(16.8) | 335(15.6) |  | 18(18.0) | 13(13.0) |  |
| Black | 11(10.3) | 287(13.4) |  | 11(11.0) | 13(13.0) |  |
| Other | 1(0.9) | 31(1.4) |  | 1(1.0) | 2(2.0) |  |
| Marital Status |  |  | 0.206 |  |  | 0.973 |
| Married | 61(57.0) | 1351(62.9) |  | 61(61.0) | 61(61.0) |  |
| Single | 17(15.9) | 366(17.0) |  | 15(15.0) | 14(14.0) |  |
| Other | 29(27.1) | 430(20.0) |  | 24(24.0) | 25(25.0) |  |
| AFP |  |  | 0.204 |  |  | 0.590 |
| Negative | 24(22.4) | 596(27.8) |  | 22(22.0) | 26(26.0) |  |
| Positive | 63(58.9) | 1075(50.1) |  | 58(58.0) | 59(59.0) |  |
| Borderline/Unknown | 20(18.7) | 476(22.2) |  | 20(20.0) | 15(15.0) |  |
| First Malignant |  |  | 0.503 |  |  | 1.000 |
| Yes | 92(86.0) | 1902(88.6) |  | 87(87.0) | 86(86.0) |  |
| No | 15(14.0) | 245(11.4) |  | 13(13.0) | 14(14.0) |  |
| Primary Tumor |  |  | 0.725 |  |  | - |
| Yes | 107(100.0) | 2130(99.2) |  | 100(100.0) | 100(100.0) |  |
| No | 0(0) | 17(0.8) |  | 0(0) | 0(0) |  |
| Neoadjuvant Therapy |  |  | 0.138 |  |  | 0.843 |
| Yes | 15(14.0) | 438(20.4) |  | 14(14.0) | 16(16.0) |  |
| No | 92(86.0) | 1709(79.6) |  | 86(86.0) | 84(84.0) |  |
| Tumor Number |  |  |  |  |  | 0.150 |
| Single | 68(63.6) | 1528(71.2) |  | 65(65.0) | 54(54.0) |  |
| Multiple | 39(36.4) | 619(28.8) |  | 35(35.0) | 46(46.0) |  |
| Tumor Size |  |  | <0.001 |  |  | 0.677 |
| ≤2cm | 3(2.8) | 473(22.0) |  | 3(3.0) | 5(5.0) |  |
| 2-5cm | 33(30.8) | 957(44.6) |  | 32(32.0) | 33(33.0) |  |
| ＞5cm | 67(62.6) | 680(31.7) |  | 62(62.0) | 61(61.0) |  |
| Unknown | 4(3.7) | 37(1.7) |  | 3(3.0) | 1(1.0) |  |
| Surgery |  |  | <0.001 |  |  | 0.632 |
| LD | 12(11.2) | 49(2.3) |  | 9(9.0) | 9(9.0) |  |
| LR | 79(73.8) | 953(44.4) |  | 75(75.0) | 69(69.0) |  |
| LT | 15(14.0) | 1136(52.9) |  | 15(15.0) | 19(19.0) |  |
| Method Unknown | 1(0.9) | 9(0.4) |  | 1(1.0) | 3(3.0) |  |
| Radiotherapy |  |  | 0.481 |  |  | 0.766 |
| Yes | 6(5.6) | 81(3.8) |  | 5(5.0) | 7(7.0) |  |
| No/Unknown | 101(94.4) | 2066(96.2) |  | 95(95.0) | 93(93.0) |  |
| Chemotherapy |  |  | 0.079 |  |  | 0.554 |
| Yes | 43(40.2) | 678(31.6) |  | 38(38.0) | 33(33.0) |  |
| No/Unknown | 64(59.8) | 1469(68.4) |  | 62(62.0) | 67(67.0) |  |
| T Stage |  |  | <0.001 |  |  | 0.531 |
| T1a | 1(0.9) | 313(14.6) |  | 1(1.0) | 1(1.0) |  |
| T1b | 35(32.7) | 749(34.9) |  | 35(35.0) | 25(25.0) |  |
| T1NOS | 2(1.9) | 18(0.8) |  | 1(1.0) | 1(1.0) |  |
| T2 | 15(14.0) | 701(32.7) |  | 15(15.0) | 17(17.0) |  |
| T3 | 25(23.4) | 176(8.2) |  | 22(22.0) | 30(30.0) |  |
| T4 | 27(25.2) | 171(8.0) |  | 24(24.0) | 26(26.0) |  |
| TX | 2(1.9) | 19(0.9) |  | 2(2.0) | 0(0) |  |
| M Stage |  |  | <0.001 |  |  | 0.794 |
| M0 | 98(91.6) | 2109(98.2) |  | 93(93.0) | 91(91.0) |  |
| M1 | 9(8.4) | 38(1.8) |  | 7(7.0) | 9(9.0) |  |
| Grade^†^ |  |  | <0.001 |  |  | 0.941 |
| G1 | 7(6.5) | 460(21.4) |  | 7(7.0) | 9(9.0) |  |
| G2 | 38(35.5) | 929(43.3) |  | 36(36.0) | 36(36.0) |  |
| G3-G4 | 45(42.1) | 371(17.3) |  | 41(41.0) | 38(38.0) |  |
| Unknown | 17(15.9) | 387(18.0) |  | 16(16.0) | 17(17.0) |  |
| Ishak Score |  |  | 0.086 |  |  | 0.978 |
| 0-4 | 14(13.1) | 285(13.3) |  | 14(14.0) | 13(13.0) |  |
| 5-6 | 22(20.6) | 648(30.2) |  | 20(20.0) | 20(20.0) |  |
| Unknown | 71(66.4) | 1214(56.5) |  | 66(66.0) | 67(67.0) |  |

HCC, Hepatocellular carcinoma; LNM, Lymph node metastasis; PSM, Propensity score matching; AFP, Alpha-fetoprotein; LD, Local destruction; LR, Liver resection; LT, Liver transplantation.

^†^G1=Well differentiated; G2=Moderately differentiated; G3-4=Poorly differentiated/Undifferentiated.

**Table S6**. Comparison between LNM and Non-LNM before and after PSM in patients with fibrolamellar carcinoma

| Factors | Before PSM | | | After PSM | | |
| --- | --- | --- | --- | --- | --- | --- |
|  | LNM  (n=41) | Non-LNM  (n=39) | P | LNM  (n=19) | Non-LNM  (n=19) | P |
| Year of Diagnosis |  |  | 0.222 |  |  | 0.515 |
| 2004-2008 | 16(39.0) | 14(35.9) |  | 7(36.8) | 6(31.6) |  |
| 2009-2013 | 8(19.5) | 14(35.9) |  | 3(15.8) | 6(31.6) |  |
| 2014-2018 | 17(41.5) | 11(28.2) |  | 9(47.4) | 7(36.8) |  |
| Age |  |  | 0.980 |  |  | - |
| ≤57 | 41(100.0) | 38(97.4) |  | 19(100.0) | 19(100.0) |  |
| 57-64 | 0(0) | 0(0) |  | 0(0) | 0(0) |  |
| 64-73 | 0(0) | 1(2.6) |  | 0(0) | 0(0) |  |
| >73 | 0(0) | 0(0) |  | 0(0) | 0(0) |  |
| Gender |  |  | 1.000 |  |  | 0.727 |
| Female | 15(36.6) | 14(35.9) |  | 7(36.8) | 5(26.3) |  |
| Male | 26(63.4) | 25(64.1) |  | 12(63.2) | 14(73.7) |  |
| Race |  |  | 0.444 |  |  | 0.444 |
| White | 35(85.4) | 31(79.5) |  | 17(89.5) | 14(73.7) |  |
| Asia-Pacific | 4(9.8) | 3(7.7) |  | 1(5.3) | 3(15.8) |  |
| Black | 2(4.9) | 5(12.8) |  | 1(5.3) | 2(10.5) |  |
| Marital Status |  |  | 0.634 |  |  | 0.565 |
| Married | 8(19.5) | 10(25.6) |  | 3(15.8) | 4(21.1) |  |
| Single | 29(70.7) | 27(69.2) |  | 15(78.9) | 15(78.9) |  |
| Other | 4(9.8) | 2(5.1) |  | 1(5.3) | 0(0) |  |
| AFP |  |  | 0.907 |  |  | 0.881 |
| Negative | 25(61.0) | 22(56.4) |  | 11(57.9) | 11(57.9) |  |
| Positive | 7(17.1) | 7(17.9) |  | 3(15.8) | 4(21.1) |  |
| Borderline/Unknown | 9(22.0) | 10(25.6) |  | 5(26.3) | 4(21.1) |  |
| First Malignant |  |  | 0.980 |  |  | - |
| Yes | 41(100.0) | 38(97.4) |  | 19(100.0) | 19(100.0) |  |
| No | 0(0) | 1(2.6) |  | 0(0) | 0(0) |  |
| Primary Tumor |  |  | - |  |  | - |
| Yes | 41(100.0) | 39(100.0) |  | 19(100.0) | 19(100.0) |  |
| No | 0(0) | 0(0) |  | 0(0) | 0(0) |  |
| Neoadjuvant Therapy |  |  | 1.000 |  |  | 0.597 |
| Yes | 4(9.8) | 4(10.3) |  | 1(5.3) | 3(15.8) |  |
| No | 37(90.2) | 35(89.7) |  | 18(94.7) | 16(84.2) |  |
| Tumor Number |  |  | 0.299 |  |  | 1.000 |
| Single | 31(75.6) | 34(87.2) |  | 14(73.7) | 15(78.9) |  |
| Multiple | 10(24.4) | 5(12.8) |  | 5(26.3) | 4(21.1) |  |
| Tumor Size |  |  | 0.004 |  |  | - |
| ≤2cm | 0(0) | 2(5.1) |  | 0(0) | 0(0) |  |
| 2-5cm | 0(0) | 5(12.8) |  | 0(0) | 0(0) |  |
| ＞5cm | 41(100.0) | 28(71.8) |  | 19(100.0) | 19(100.0) |  |
| Unknown | 0(0) | 4(10.3) |  | 0(0) | 0(0) |  |
| Surgery |  |  | 0.572 |  |  | 0.337 |
| LR | 36(87.8) | 34(87.2) |  | 18(94.7) | 15(78.9) |  |
| LT | 4(9.8) | 5(12.8) |  | 1(5.3) | 4(21.1) |  |
| Method Unknown | 1(2.4) | 0(0) |  | 0(0) | 0(0) |  |
| Radiotherapy |  |  | 0.572 |  |  | 1.000 |
| Yes | 1(2.4) | 3(7.7) |  | 1(5.3) | 1(5.3) |  |
| No/Unknown | 40(97.6) | 36(92.3) |  | 18(94.7) | 18(94.7) |  |
| Chemotherapy |  |  | 0.005 |  |  | 1.000 |
| Yes | 22(53.7) | 8(20.5) |  | 7(36.8) | 6(31.6) |  |
| No/Unknown | 19(46.3) | 31(79.5) |  | 12(63.2) | 13(68.4) |  |
| T Stage |  |  | 0.226 |  |  | 0.307 |
| T1b | 14(34.1) | 20(51.3) |  | 4(21.1) | 9(47.4) |  |
| T1NOS | 0(0) | 2(5.1) |  | 0(0) | 0(0) |  |
| T2 | 8(19.5) | 5(12.8) |  | 5(26.3) | 2(10.5) |  |
| T3 | 8(19.5) | 4(10.3) |  | 4(21.1) | 4(21.1) |  |
| T4 | 11(26.8) | 7(17.9) |  | 6(31.6) | 4(21.1) |  |
| TX | 0(0) | 1(2.6) |  | 0(0) | 0(0) |  |
| M Stage |  |  | 0.029 |  |  | 1.000 |
| M0 | 29(70.7) | 36(92.3) |  | 18(94.7) | 17(89.5) |  |
| M1 | 12(29.3) | 3(7.7) |  | 1(5.3) | 2(10.5) |  |
| Grade^†^ |  |  | 0.392 |  |  | 0.621 |
| G1 | 4(9.8) | 4(10.3) |  | 2(10.5) | 4(21.1) |  |
| G2 | 17(41.5) | 14(35.9) |  | 9(47.4) | 9(47.4) |  |
| G3-G4 | 6(14.6) | 2(5.1) |  | 0(0) | 0(0) |  |
| Unknown | 14(34.1) | 19(48.7) |  | 8(42.1) | 6(31.6) |  |
| Ishak Score |  |  | 0.355 |  |  | 0.269 |
| 0-4 | 7(17.1) | 11(28.2) |  | 3(15.8) | 7(36.8) |  |
| Unknown | 34(82.9) | 28(71.8) |  | 16(84.2) | 12(63.2) |  |

LNM, Lymph node metastasis; PSM, Propensity score matching; AFP, Alpha-fetoprotein; LD, Local destruction; LR, Liver resection; LT, Liver transplantation.

^†^G1=Well differentiated; G2=Moderately differentiated; G3-4=Poorly differentiated/Undifferentiated.

**Table S7**. Case series of LT recipients with rare pathological subtypes of HCC

| Year of Diagnosis | Patient’s ID | Pathological  Subtype | Gender | Age  (years) | Race | AFP | Cirrhosis | TNM Stage | | | Size  (cm) | Multiple Tumor | Neoadjuvant Therapy | Che | Rad | Milan Criteria | Time  (months) | Alive |
| --- | --- | --- | --- | --- | --- | --- | --- | --- | --- | --- | --- | --- | --- | --- | --- | --- | --- | --- |
| 2004 | 43813563 | Fibrolamellar | M | 39 | W | + | UNK | T4 | N0 | M0 | 11.0 | N | N | Y | N | Beyond | 18 | N |
| 2005 | 57502789 | Fibrolamellar | F | 23 | W | + | UNK | T3 | NX | M0 | 17.5 | Y | N | N | N | Beyond | 20 | N |
| 2007 | 23833940 | Fibrolamellar | F | 15 | W | + | UNK | T3 | N1 | M1 | 20.0 | Y | N | Y | N | Beyond | 136 | Y |
| 2007 | 35709859 | Fibrolamellar | F | 31 | W | UNK | UNK | T3 | N1 | M0 | 7.8 | Y | N | Y | N | Beyond | 29 | N |
| 2010 | 66355396 | Fibrolamellar | F | 55 | W | - | UNK | T1b | NX | M0 | 5.5 | N | N | Y | N | Beyond | 92 | N |
| 2011 | 28413505 | Fibrolamellar | M | 36 | W | - | UNK | TX | N0 | M0 | UNK | Y | Y | N | N | UNK | 90 | Y |
| 2012 | 45028505 | Fibrolamellar | M | 9 | W | - | - | T4 | N1 | M0 | 14.0 | Y | Y | Y | N | Beyond | 80 | Y |
| 2012 | 45124588 | Fibrolamellar | M | 16 | W | - | UNK | T3 | N0 | M0 | 16.8 | Y | Y | Y | N | Beyond | 48 | N |
| 2013 | 30581403 | Fibrolamellar | F | 13 | B | - | UNK | T4 | NX | M0 | 17.3 | Y | Y | Y | N | Beyond | 59 | Y |
| 2013 | 36147820 | Fibrolamellar | M | 11 | W | + | - | T4 | N0 | M0 | 12.7 | N | Y | Y | N | Beyond | 34 | N |
| 2013 | 36152673 | Fibrolamellar | M | 53 | W | + | UNK | T1b | NX | M0 | 3.5 | N | Y | Y | N | Beyond | 66 | Y |
| 2014 | 30480891 | Fibrolamellar | M | 21 | B | - | UNK | T1b | N0 | M0 | 7.5 | N | N | N | N | Beyond | 53 | Y |
| 2014 | 45341855 | Fibrolamellar | M | 18 | W | - | UNK | T4 | N1 | M1 | 31.0 | N | N | Y | N | Beyond | 37 | N |
| 2005 | 6045421 | Scirrhous | M | 52 | W | - | UNK | T2 | NX | M0 | 2.9 | Y | Y | Y | N | Beyond | 164 | Y |
| 2006 | 1035350 | Scirrhous | M | 65 | W | UNK | + | T2 | N0 | M0 | 3.0 | Y | N | N | N | Within | 58 | N |
| 2006 | 44144042 | Scirrhous | M | 59 | W | UNK | + | T2 | NX | M0 | 1.7 | Y | N | N | N | UNK | 42 | N |
| 2006 | 66025156 | Scirrhous | M | 55 | W | UNK | UNK | T2 | N0 | M0 | 1.6 | Y | N | N | N | Within | 150 | Y |
| 2011 | 66533302 | Scirrhous | M | 57 | W | - | UNK | T2 | N0 | M0 | 2.6 | N | Y | Y | N | Beyond | 90 | Y |
| 2016 | 30572950 | Scirrhous | M | 58 | W | - | + | T1a | N0 | M0 | 1.2 | N | N | N | N | Beyond | 31 | Y |
| 2010 | 60745777 | Spindle Cell | M | 59 | W | + | UNK | T2 | N0 | M0 | 2.6 | Y | Y | Y | N | UNK | 94 | N |
| 2017 | 46006680 | Spindle Cell | M | 52 | W | + | - | T1b | NX | M0 | 2.9 | N | N | N | N | Within | 9 | Y |
| 2004 | 6024747 | Clear Cell | M | 59 | W | UNK | UNK | T2 | NX | M0 | 2.2 | Y | N | N | N | UNK | 177 | Y |
| 2004 | 35511316 | Clear Cell | F | 65 | W | UNK | UNK | T2 | N0 | M0 | 1.2 | Y | N | N | N | Within | 9 | N |
| 2004 | 43923186 | Clear Cell | M | 55 | AP | + | UNK | T1b | NX | M0 | 3.6 | N | N | N | N | Within | 158 | N |
| 2005 | 1003316 | Clear Cell | M | 60 | W | + | + | T2 | NX | M0 | 2.0 | Y | Y | Y | N | Beyond | 156 | Y |
| 2005 | 44079144 | Clear Cell | M | 62 | W | UNK | + | T2 | NX | M0 | 0.9 | Y | N | N | N | Within | 165 | Y |
| 2005 | 75047993 | Clear Cell | M | 51 | B | UNK | UNK | T2 | NX | M0 | 0.9 | Y | N | N | N | UNK | 48 | N |
| 2006 | 44057440 | Clear Cell | F | 61 | W | - | UNK | T1b | N0 | M0 | 3.6 | N | N | N | N | Within | 124 | N |
| 2007 | 60587860 | Clear Cell | M | 59 | W | + | UNK | T2 | NX | M0 | 4.7 | Y | Y | Y | N | Beyond | 5 | N |
| 2009 | 57631544 | Clear Cell | M | 63 | B | - | UNK | T1b | N0 | M0 | 5.0 | N | Y | Y | N | Within | 82 | N |
| 2010 | 60754997 | Clear Cell | M | 66 | W | UNK | UNK | T2 | NX | M0 | 1.2 | Y | N | N | N | Within | 94 | N |
| 2012 | 66604330 | Clear Cell | M | 59 | W | + | + | T1a | NX | M0 | 1.5 | N | N | N | N | Within | 79 | Y |
| 2017 | 58022805 | Clear Cell | F | 64 | W | - | - | T2 | N0 | M0 | 5.9 | N | N | Y | N | Beyond | 14 | Y |
| 2018 | 67186179 | Clear Cell | F | 59 | B | - | + | T2 | N0 | M0 | 2.4 | N | N | N | N | Beyond | 11 | Y |
| 2018 | 67268293 | Clear Cell | M | 59 | W | - | UNK | T2 | NX | M0 | 4.5 | N | N | N | N | Beyond | 7 | Y |

LT, Liver transplantation; HCC, Hepatocellular carcinoma; AFP, Alpha-fetoprotein; Che, Chemotherapy; Rad, Radiotherapy; M, Male; F, Female; W, White; B, Black; AP, Asia-Pacific; UNK, Unknown; Y, Yes; N, No.

**Table S8**. Baseline characteristics of patients with rare pathological subtypes of HCC in the training and validation sets

| Factors | Training Set (n=476) | Validation Set (n=476) |
| --- | --- | --- |
| Year of Diagnosis |  |  |
| 2004-2008 | 138(29.0) | 155(32.6) |
| 2009-2013 | 158(33.2) | 161(33.8) |
| 2014-2018 | 180(37.8) | 160(33.6) |
| Age |  |  |
| ≤57 | 183(38.4) | 171(35.9) |
| 57-64 | 78(16.4) | 85(17.9) |
| 64-73 | 105(22.1) | 106(22.3) |
| >73 | 110(23.1) | 114(23.9) |
| Gender |  |  |
| Female | 181(38.0) | 170(35.7) |
| Male | 295(62.0) | 306(64.3) |
| Race |  |  |
| White | 338(71.0) | 342(71.8) |
| Asia-Pacific | 77(16.2) | 73(15.3) |
| Black | 57(12.0) | 54(11.3) |
| Other | 4(0.8) | 7(1.5) |
| Marital Status |  |  |
| Married | 236(49.6) | 251(52.7) |
| Single | 137(28.8) | 122(25.6) |
| Other | 103(21.6) | 103(21.6) |
| AFP |  |  |
| Negative | 140(29.4) | 136(28.6) |
| Positive | 209(43.9) | 229(48.1) |
| Borderline/Unknown | 127(26.7) | 111(23.3) |
| First Malignant |  |  |
| Yes | 406(85.3) | 400(84.0) |
| No | 70(14.7) | 76(16.0) |
| Primary Tumor |  |  |
| Yes | 474(99.6) | 473(99.4) |
| No | 2(0.4) | 3(0.6) |
| Neoadjuvant Therapy |  |  |
| Yes | 18(3.8) | 11(2.3) |
| No | 458(96.2) | 465(97.7) |
| Tumor Number |  |  |
| Single | 334(70.2) | 333(70.0) |
| Multiple | 142(29.8) | 143(30.0) |
| Tumor Size |  |  |
| ≤2cm | 23(4.8) | 33(6.9) |
| 2-5cm | 107(22.5) | 117(24.6) |
| ＞5cm | 270(56.7) | 257(54.0) |
| Unknown | 76(16.0) | 69(14.5) |
| Surgery |  |  |
| None | 263(55.3) | 280(58.8) |
| LD | 31(6.5) | 29(6.1) |
| LR | 165(34.7) | 144(30.3) |
| LT | 15(3.2) | 20(4.2) |
| Method Unknown | 2(0.4) | 3(0.6) |
| Radiotherapy |  |  |
| Yes | 34(7.1) | 46(9.7) |
| No/Unknown | 442(92.9) | 430(90.3) |
| Chemotherapy |  |  |
| Yes | 176(37.0) | 158(33.2) |
| No/Unknown | 300(63.0) | 318(66.8) |
| T Stage |  |  |
| T1-T2 | 267(56.1) | 292(61.3) |
| T3-T4 | 151(31.7) | 139(29.2) |
| TX | 58(12.2) | 45(9.5) |
| N Stage |  |  |
| N0 | 43(9.0) | 39(8.2) |
| N1 | 28(5.9) | 15(3.2) |
| NX | 405(85.1) | 422(88.7) |
| M Stage |  |  |
| M0 | 386(81.1) | 379(79.6) |
| M1 | 90(18.9) | 97(20.4) |
| Grade^†^ |  |  |
| G1 | 64(13.4) | 50(10.5) |
| G2 | 146(30.7) | 124(26.1) |
| G3-G4 | 85(17.9) | 71(14.9) |
| Unknown | 181(38.0) | 231(48.5) |
| Ishak Score |  |  |
| 0-4 | 59(12.4) | 59(12.4) |
| 5-6 | 46(9.7) | 47(9.9) |
| Unknown | 371(77.9) | 370(77.7) |
| Pathological Subtype |  |  |
| Fibrolamellar | 119(25.0) | 122(25.6) |
| Scirrhous | 47(9.9) | 35(7.4) |
| Spindle Cell | 26(5.5) | 35(7.4) |
| Clear Cell | 279(58.6) | 272(57.1) |
| Pleomorphic | 5(1.1) | 12(2.5) |

HCC, Hepatocellular carcinoma; AFP, Alpha-fetoprotein; LD, Local destruction; LR, Liver resection; LT, Liver transplantation.

^†^G1=Well differentiated; G2=Moderately differentiated; G3-4=Poorly differentiated/Undifferentiated.

**Table S9**. Competing risk survival analyses of patients with rare pathological subtypes of HCC

| Factors | No. of Patients  (n=476) | Univariable | | Multivariate | |
| --- | --- | --- | --- | --- | --- |
|  |  | P-CSD | P-OCSD | SHR (95%CI) | P-CSD |
| Year of Diagnosis |  | 0.604 | 0.158 |  |  |
| 2004-2008 | 138(29.0) |  |  | Reference |  |
| 2009-2013 | 158(33.2) |  |  | 1.154(0.859-1.552) | 0.340 |
| 2014-2018 | 180(37.8) |  |  | 0.830(0.598-1.152) | 0.270 |
| Age |  | 0.456 | 0.006 |  |  |
| ≤57 | 183(38.4) |  |  | Reference |  |
| 57-64 | 78(16.4) |  |  | 1.181(0.770-1.811) | 0.450 |
| 64-73 | 105(22.1) |  |  | 1.185(0.815-1.723) | 0.370 |
| >73 | 110(23.1) |  |  | 1.002(0.636-1.577) | 0.990 |
| Gender |  | 0.116 | 0.045 |  |  |
| Female | 181(38.0) |  |  | Reference |  |
| Male | 295(62.0) |  |  | 0.923(0.698-1.221) | 0.580 |
| Race |  | 0.621 | 0.653 |  |  |
| White | 338(71.0) |  |  | Reference |  |
| Asia-Pacific | 77(16.2) |  |  | 0.918(0.642-1.312) | 0.640 |
| Black | 57(12.0) |  |  | 0.982(0.675-1.430) | 0.930 |
| Other | 4(0.8) |  |  | 0.328(0.037-2.894) | 0.320 |
| Marital Status |  | 0.010 | 0.115 |  |  |
| Married | 236(49.6) |  |  | Reference |  |
| Single | 137(28.8) |  |  | 0.922(0.655-1.298) | 0.640 |
| Other | 103(21.6) |  |  | 1.075(0.765-1.511) | 0.680 |
| AFP |  | <0.001 | 0.175 |  |  |
| Negative | 140(29.4) |  |  | Reference |  |
| Positive | 209(43.9) |  |  | 1.425(1.034-1.966) | 0.031 |
| Borderline/Unknown | 127(26.7) |  |  | 1.164(0.800-1.692) | 0.430 |
| First Malignant |  | 0.142 | <0.001 |  |  |
| Yes | 406(85.3) |  |  | Reference |  |
| No | 70(14.7) |  |  | 0.566(0.360-0.889) | 0.014 |
| Primary Tumor |  | 0.305 | 0.711 |  |  |
| Yes | 474(99.6) |  |  | Reference |  |
| No | 2(0.4) |  |  | 2.599(0.734-9.205) | 0.140 |
| Neoadjuvant Therapy |  | 0.033 | 0.405 |  |  |
| Yes | 18(3.8) |  |  | Reference |  |
| No | 458(96.2) |  |  | 1.121(0.540-2.325) | 0.760 |
| Tumor Number |  | <0.001 | 0.517 |  |  |
| Single | 334(70.2) |  |  | Reference |  |
| Multiple | 142(29.8) |  |  | 1.023(0.761-1.374) | 0.880 |
| Tumor Size |  | <0.001 | 0.423 |  |  |
| ≤2cm | 23(4.8) |  |  | Reference |  |
| 2-5cm | 107(22.5) |  |  | 0.663(0.376-1.170) | 0.160 |
| ＞5cm | 270(56.7) |  |  | 0.759(0.409-1.406) | 0.380 |
| Unknown | 76(16.0) |  |  | 0.988(0.489-1.996) | 0.970 |
| Surgery |  | <0.001 | 0.230 |  |  |
| None | 263(55.3) |  |  | Reference |  |
| LD | 31(6.5) |  |  | 0.450(0.280-0.724) | 0.001 |
| LR | 165(34.7) |  |  | 0.322(0.216-0.480) | <0.001 |
| LT | 15(3.2) |  |  | 0.215(0.076-0.609) | 0.004 |
| Method Unknown | 2(0.4) |  |  | 0.741(0.140-3.928) | 0.730 |
| Radiotherapy |  | 0.122 | 0.574 |  |  |
| Yes | 34(7.1) |  |  | Reference |  |
| No/Unknown | 442(92.9) |  |  | 1.171(0.794-1.729) | 0.430 |
| Chemotherapy |  | 0.045 | 0.242 |  |  |
| Yes | 176(37.0) |  |  | Reference |  |
| No/Unknown | 300(63.0) |  |  | 1.139(0.854-1.517) | 0.380 |
| T Stage |  | <0.001 | 0.976 |  |  |
| T1-T2 | 267(56.1) |  |  | Reference |  |
| T3-T4 | 151(31.7) |  |  | 1.774(1.230-2.559) | 0.002 |
| TX | 58(12.2) |  |  | 1.578(0.909-2.742) | 0.110 |
| N Stage |  | <0.001 | 0.157 |  |  |
| N0 | 43(9.0) |  |  | Reference |  |
| N1 | 28(5.9) |  |  | 1.105(0.547-2.230) | 0.780 |
| NX | 405(85.1) |  |  | 0.779(0.452-1.343) | 0.370 |
| M Stage |  | <0.001 | 0.115 |  |  |
| M0 | 386(81.1) |  |  | Reference |  |
| M1 | 90(18.9) |  |  | 1.539(1.106-2.144) | 0.011 |
| Grade^†^ |  | <0.001 | 0.112 |  |  |
| G1 | 64(13.4) |  |  | Reference |  |
| G2 | 146(30.7) |  |  | 0.770(0.530-1.118) | 0.170 |
| G3-G4 | 85(17.9) |  |  | 1.097(0.710-1.695) | 0.680 |
| Unknown | 181(38.0) |  |  | 0.833(0.578-1.202) | 0.330 |
| Ishak Score |  | 0.007 | 0.536 |  |  |
| 0-4 | 59(12.4) |  |  | Reference |  |
| 5-6 | 46(9.7) |  |  | 0.808(0.493-1.323) | 0.400 |
| Unknown | 371(77.9) |  |  | 0.923(0.652-1.306) | 0.650 |
| Pathological Subtype |  | <0.001 | 0.055 |  |  |
| Fibrolamellar | 119(25.0) |  |  | Reference |  |
| Scirrhous | 47(9.9) |  |  | 1.061(0.621-1.812) | 0.830 |
| Spindle Cell | 26(5.5) |  |  | 2.702(1.280-5.707) | 0.009 |
| Clear Cell | 279(58.6) |  |  | 1.091(0.703-1.695) | 0.700 |
| Pleomorphic | 5(1.1) |  |  | 1.300(0.363-4.657) | 0.690 |

HCC, Hepatocellular carcinoma; CSD, Cancer-specific death; OCSD, Other cause-specific death; SHR, Subdistribution hazard ratio; CI, Confidence interval; AFP, Alpha-fetoprotein; LD, Local destruction; LR, Liver resection; LT, Liver transplantation.

^†^G1=Well differentiated; G2=Moderately differentiated; G3-4=Poorly differentiated/Undifferentiated.

**Table S10**. Cumulative incidence of CSD and OCSD of patients with rare pathological subtypes of HCC

| Factors | CSD | | | OCSD | | |
| --- | --- | --- | --- | --- | --- | --- |
|  | 1year-CI | 3year-CI | 5year-CI | 1year-CI | 3year-CI | 5year-CI |
| Year of Diagnosis |  |  |  |  |  |  |
| 2004-2008 | 0.356 | 0.532 | 0.612 | 0.065 | 0.095 | 0.117 |
| 2009-2013 | 0.340 | 0.568 | 0.641 | 0.032 | 0.058 | 0.058 |
| 2014-2018 | 0.372 | 0.520 | - | 0.049 | 0.080 | - |
| Age |  |  |  |  |  |  |
| ≤57 | 0.291 | 0.489 | 0.596 | 0.022 | 0.047 | 0.047 |
| 57-64 | 0.371 | 0.527 | 0.621 | 0.027 | 0.054 | 0.072 |
| 64-73 | 0.383 | 0.567 | 0.622 | 0.060 | 0.072 | 0.096 |
| >73 | 0.436 | 0.632 | 0.669 | 0.096 | 0.153 | 0.153 |
| Gender |  |  |  |  |  |  |
| Female | 0.341 | 0.611 | 0.684 | 0.040 | 0.046 | 0.055 |
| Male | 0.367 | 0.507 | 0.586 | 0.053 | 0.095 | 0.104 |
| Race |  |  |  |  |  |  |
| White | 0.349 | 0.544 | 0.617 | 0.046 | 0.073 | 0.084 |
| Asia-Pacific | 0.382 | 0.566 | 0.629 | 0.043 | 0.076 | 0.076 |
| Black | 0.378 | 0.540 | 0.655 | 0.053 | 0.091 | 0.091 |
| Other | 0.250 | 0.250 | 0.250 | 0.375 | 0.375 | 0.375 |
| Marital Status |  |  |  |  |  |  |
| Married | 0.352 | 0.532 | 0.603 | 0.057 | 0.100 | 0.106 |
| Single | 0.287 | 0.487 | 0.603 | 0.007 | 0.024 | 0.035 |
| Other | 0.461 | 0.652 | 0.692 | 0.082 | 0.093 | 0.107 |
| AFP |  |  |  |  |  |  |
| Negative | 0.249 | 0.402 | 0.474 | 0.022 | 0.047 | 0.047 |
| Positive | 0.418 | 0.647 | 0.719 | 0.064 | 0.086 | 0.092 |
| Borderline/Unknown | 0.374 | 0.528 | 0.618 | 0.051 | 0.099 | 0.120 |
| First Malignant |  |  |  |  |  |  |
| Yes | 0.360 | 0.550 | 0.635 | 0.033 | 0.056 | 0.062 |
| No | 0.338 | 0.511 | 0.533 | 0.140 | 0.207 | 0.229 |
| Primary Tumor |  |  |  |  |  |  |
| Yes | 0.358 | 0.542 | 0.619 | 0.048 | 0.078 | 0.086 |
| No | 0 | - | - | 0 | - | - |
| Neoadjuvant Therapy |  |  |  |  |  |  |
| Yes | 0.111 | 0.278 | 0.412 | 0 | 0 | 0 |
| No | 0.367 | 0.556 | 0.630 | 0.050 | 0.081 | 0.089 |
| Tumor Number |  |  |  |  |  |  |
| Single | 0.317 | 0.484 | 0.551 | 0.044 | 0.083 | 0.091 |
| Multiple | 0.445 | 0.679 | 0.779 | 0.057 | 0.065 | 0.075 |
| Tumor Size |  |  |  |  |  |  |
| ≤2cm | 0.348 | 0.441 | 0.543 | 0 | 0 | 0.102 |
| 2-5cm | 0.192 | 0.348 | 0.444 | 0.048 | 0.102 | 0.113 |
| ＞5cm | 0.348 | 0.572 | 0.655 | 0.042 | 0.063 | 0.063 |
| Unknown | 0.635 | 0.776 | 0.791 | 0.084 | 0.115 | 0.115 |
| Surgery |  |  |  |  |  |  |
| None | 0.570 | 0.768 | 0.814 | 0.079 | 0.116 | 0.116 |
| LD | 0.102 | 0.286 | 0.461 | 0.033 | 0.104 | 0.145 |
| LR | 0.089 | 0.284 | 0.383 | 0.006 | 0.208 | 0.029 |
| LT | 0.133 | 0.200 | 0.408 | 0 | 0 | 0.067 |
| Method Unknown | 0.500 | 0.500 | 0.500 | 0 | 0 | 0 |
| Radiotherapy |  |  |  |  |  |  |
| Yes | 0.360 | 0.736 | - | 0.066 | 0.104 | - |
| No/Unknown | 0.321 | 0.533 | 0.610 | 0.047 | 0.075 | 0.084 |
| Chemotherapy |  |  |  |  |  |  |
| Yes | 0.350 | 0.602 | 0.698 | 0.040 | 0.072 | 0.072 |
| No/Unknown | 0.359 | 0.506 | 0.570 | 0.052 | 0.080 | 0.094 |
| T Stage |  |  |  |  |  |  |
| T1-T2 | 0.246 | 0.414 | 0.480 | 0.039 | 0.077 | 0.092 |
| T3-T4 | 0.458 | 0.707 | 0.827 | 0.055 | 0.078 | 0.078 |
| TX | 0.610 | 0.746 | 0.772 | 0.072 | 0.072 | 0.072 |
| N Stage |  |  |  |  |  |  |
| N0 | 0.119 | 0.239 | 0.367 | 0 | 0 | 0 |
| N1 | 0.073 | 0.315 | 0.491 | 0 | 0 | 0 |
| NX | 0.401 | 0.592 | 0.657 | 0.056 | 0.090 | 0.100 |
| M Stage |  |  |  |  |  |  |
| M0 | 0.292 | 0.482 | 0.567 | 0.051 | 0.081 | 0.920 |
| M1 | 0.634 | 0.805 | 0.849 | 0.035 | 0.058 | 0.058 |
| Grade^†^ |  |  |  |  |  |  |
| G1 | 0.268 | 0.461 | 0.571 | 0.016 | 0.016 | 0.016 |
| G2 | 0.221 | 0.439 | 0.531 | 0.042 | 0.065 | 0.083 |
| G3-G4 | 0.473 | 0.655 | 0.740 | 0.050 | 0.066 | 0.066 |
| Unknown | 0.444 | 0.609 | 0.660 | 0.063 | 0.115 | 0.123 |
| Ishak Score |  |  |  |  |  |  |
| 0-4 | 0.156 | 0.392 | 0.450 | 0.018 | 0.018 | 0.074 |
| 5-6 | 0.224 | 0.484 | 0.540 | 0.045 | 0.091 | 0.116 |
| Unknown | 0.406 | 0.576 | 0.657 | 0.053 | 0.084 | 0.084 |
| Pathological Subtype |  |  |  |  |  |  |
| Fibrolamellar | 0.217 | 0.413 | 0.514 | 0 | 0.020 | 0.033 |
| Scirrhous | 0.440 | 0.557 | 0.641 | 0.066 | 0.066 | 0.093 |
| Spindle Cell | 0.865 | - | - | 0.040 | - | - |
| Clear Cell | 0.357 | 0.563 | 0.635 | 0.067 | 0.103 | 0.108 |
| Pleomorphic | 0.400 | 0.600 | - | 0 | 0.200 | - |

HCC, Hepatocellular carcinoma; CSD, Cancer-specific death; OCSD, Other cause-specific death; CI, Cumulative incidence; AFP, Alpha-fetoprotein; LD, Local destruction; LR, Liver resection; LT, Liver transplantation.

^†^G1=Well differentiated; G2=Moderately differentiated; G3-4=Poorly differentiated/Undifferentiated.
